# Micro- and nanoplastic exposure, immune cell activation, and lung function in young adults

**DOI:** 10.1186/s43591-026-00189-2

**Published:** 2026-05-07

**Authors:** Amanda M. Durkin, Tim L. P. Skrabanja, Ulrike Gehring, Runyu Zou, Virissa Lenters, Gerard H. Koppelman, Judith M. Vonk, Nienke Vrisekoop, Roel Vermeulen

**Affiliations:** 1 Institute for Risk Assessment Sciences, Utrecht University, Utrecht, The Netherlands; 2 Department of Global Public Health & Bioethics, Julius Center for Health Sciences and Primary Care, University Medical Centre Utrecht, Utrecht, The Netherlands; 3 Department of Respiratory Medicine and Center for Translational Immunology, University Medical Center Utrecht, Utrecht, The Netherlands; 4 Amsterdam Institute for Life and Environment, Vrije Universiteit Amsterdam, Amsterdam, The Netherlands; 5 Department of Pediatric Pulmonology and Pediatric Allergology, Beatrix Children’s Hospital, University Medical Center Groningen, University of Groningen, Groningen, The Netherlands; 6 Groningen Research Institute for Asthma and COPD, University Medical Center Groningen, University of Groningen, Groningen, The Netherlands; 7 Department of Epidemiology, University Medical Center Groningen, University of Groningen, Groningen, The Netherlands

**Keywords:** Microplastics, Lung function, Innate immunity, Epidemiology

## Abstract

**Rationale:**

Micro- and nanoplastics, an emerging public health concern, enter the human body through inhalation and ingestion. The potential for this exposure to induce a pro-inflammatory immune response or impair lung function remains unknown.

**Objectives:**

This study aimed to estimate exposure to micro- and nanoplastics in young adults and investigate associations with immune activation and lung function.

**Methods:**

We performed a cross-sectional study among 100 participants of the Dutch PIAMA (Prevention and Incidence of Asthma and Mite Allergy) cohort during the 25-year follow-up. Micro- and nanoplastic concentrations in blood were quantified with pyrolysis gas chromatography mass spectrometry. Innate immune cell activation was assessed with flow cytometry by measuring CD11b, CD62L, and CD10 expresssion on neutrophils as well as CD11b expression on monocytes and eosinophils. Lung function was measured by spirometry. Linear regression analysis was used to assess associations of micro- and nanoplastic exposure with selected immune markers and lung function parameters.

**Measurements and main results:**

Micro- and nanoplastics were detected in all samples with a median total concentration of 427.5 ng/mL. Higher total micro- and nanoplastic concentrations were associated with higher expression of neutrophil, eosinophil, and monocyte CD11b, and neutrophil CD10. Higher polyvinyl chloride concentrations were associated with higher expression of eosinophil CD11b. No associations were found between micro- and nanoplastic exposure and lung function.

**Conclusions:**

These findings suggest that micro- and nanoplastics may be associated with health perturbations in young adults. Further research is warranted to confirm these observations in larger, more diverse populations.

**Supplementary Information:**

The online version contains supplementary material available at 10.1186/s43591-026-00189-2.

## Graphical Abstract

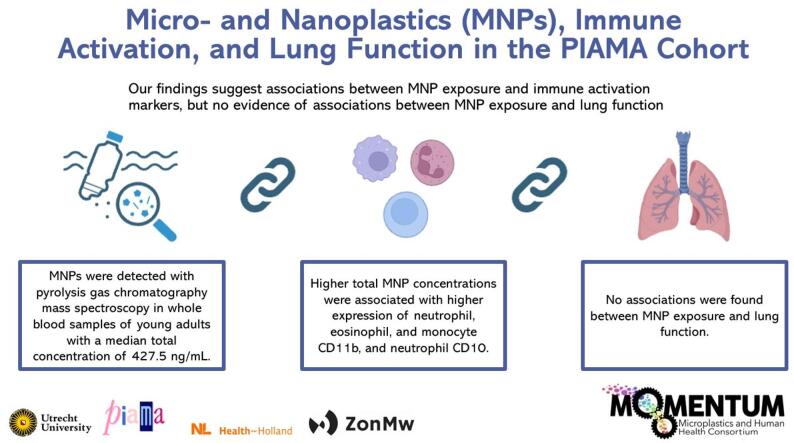

## Introduction

Micro- and nanoplastics (MNPs) are an emerging public health concern. Microplastics (MPs), defined as plastic particles with a diameter of 5 mm or less, and nanoplastics (NPs), plastic particles smaller than 1 μm, consist of polymers combined with both intentionally and non-intentionally added chemicals [1]. While it is certain that humans are exposed to MNPs, there is limited evidence about the extent of this exposure and consequent health effects [2, 3]. MNPs can be inhaled through the nose or mouth, enter the respiratory tract, and reach the lungs or they can be ingested with food and beverages [3]. Once inhaled or ingested, MNPs may translocate across membranes and enter the bloodstream [4, 5]. Recent advances in analytical methodologies now allow for measurement of MNPs in human blood [6, 7] providing a possibility to quantify internal MNP doses.

When MNPs enter the body, the immune system responds to these foreign particles [8, 9]. As with other exogenous agents, e.g., air pollutants [10], MNPs may trigger a pro-inflammatory immune response. In vitro studies have shown that neutrophils phagocytose MPs, leading to 50–95% neutrophil cell death [11]. Further, various in vitro experiments in murine and fish models indicate that MNPs impair immune function by disrupting signaling pathways and altering immune homeostasis, suggesting that similar effects may be relevant in humans [8, 9, 11, 12].

Recent studies have reported the presence of MPs in lung tissue [13–16], bronchoalveolar lavage fluid (BALF) [17–19], and sputum [20]. To date, only one study has investigated the association between MPs quantified in BALF and lung function. Conducted in 44 patients with clinical indication for bronchoscopy, this study reported an association between higher levels of MPs in BALF and lower forced expiratory volume in 1 s (FEV_1_) as well as forced vital capacity (FVC) [17]. Additionally, MP fibers have been associated with interstitial and bronchial inflammation in flock workers when present at high airborne concentrations [21, 22].

The limited number of studies of MNPs and the small sample sizes of these studies are due, in part, to a lack of comprehensive exposure assessment approaches [17–19]. While inhalation is considered a major exposure route, there is little information about how MNPs affect lung health or immune cell function. Research is needed to understand the potential long-term health consequences of inhaled MNPs. In this study, we measured MNP mass concentrations in whole blood of young adults to estimate their exposure to MNPs and examined cross-sectional associations between MNP exposure and (1) immune cell activation, assessed via markers of neutrophils, monocytes and eosinophils, and (2) lung function, as measured by FEV_1_ and FVC.

## Methods

### Study design and population

This study was performed within the population-based prospective PIAMA (Prevention and Incidence of Asthma and Mite Allergy) birth cohort study in the Netherlands, described elsewhere in detail [23]. The baseline population consisted of 3,963 newborns from communities in the North, West, and Central regions of the Netherlands. Since their birth in 1996/97, participants have been followed through repeated questionnaires that addressed demographic, lifestyle, household, and health characteristics. Questionnaires were completed by parents (until participants reached age 17) and by the participants themselves (from age 11). Medical examinations including spirometry and anthropometry were conducted at several ages in subgroups (see supplement for more details). During the most recent medical examination (May–October 2023), participants were invited to provide blood samples for MNP measurements. All participants signed an informed consent under the rules and legislation in place within the Netherlands and maintained by the Medical Ethical Committee of the University Medical Center Utrecht. Rolling recruitment was conducted with a target sample size of 100. A total of 106 participants agreed to participate in this study, and 100 were included in the final analysis (Figure S1). Participants were excluded if the blood collection procedure was not followed (*n* = 4) or if they had MNP values which were deemed likely outliers (*n* = 2).

### MNP exposure assessment

Whole blood was collected according to the plastic-free sampling procedure described by Leslie et al. [6]. In brief, venipuncture was performed to obtain whole blood, collected in 10 mL glass heparinized vacutainer tubes (BD Biosciences, Plymouth, UK) and stored at -20 °C until analysis. The mass concentrations of seven plastic polymers, including polyethylene (PE), polyethylene terephthalate (PET), poly(methyl methacrylate) (PMMA), polypropylene (PP), polystyrene (PS), polyvinyl chloride (PVC), and polyamide 6.6 (PA6.6) in whole blood were quantified using pyrolysis gas chromatography coupled with mass spectrometry (Py-GC-MS).

The analysis were performed at the Amsterdam Institute for Life and Environment of the Vrije Universiteit Amsterdam, following the previously published procedure [24]. Following Proteinase K digestion in TRIS buffer (0.5% SDS), samples were incubated at 60 °C and filtered through a 0.7 μm glass microfiber filter using a custom-made filtration assembly. Any residual organic matter was removed with 30% H₂O₂. All samples were filtered without clogging, demonstrating effective reduction of organic matrix components during digestion. The filter was then placed in a pyrolysis cup and 25 ng of poly(4-fluoro) styrene was added as an internal standard. Quantitative pyrolysis was performed using a multi-shot pyrolizer fitted with an auto-shot sampler in full scan mode. The particle size range measured was approximately 0.7 –514 μm due to the filtration step prior to analysis and the inner diameter of the needles used for the blood draw.

To accurately quantify plastic polymers in the samples, a calibration mixture (excluding PA6.6) was used to prepare calibration standards, which were analyzed with each batch of blood samples as previously described by Nardella et al. [24]. For each polymer, one indicator compound was selected for quantification. Sample analysis was performed in batches consisting of calibration standards, spiked quality control (QC) samples, blanks, and study samples. The recoveries of the quantitation compounds for the QCs analysed in the batch analysis (*n* = 15) ranged from 52 to 102% and the %RSDs from 14 to 44%. Polymer-specific recoveries are provided in Nardella et al. (2025, Table 2). PA6.6 was quantified using a calibration curve prepared from particles generated with the MOMENTUM (Microplastics and Human Health) consortium using five calibration standards [25] and, therefore, the results for PA6.6 should be considered indicative values. More information about sample analysis can be found in the supplement and all relevant method development is described in Nardella et al. [24].

**Table 1:** Characteristics of the study population (*n* = 100)

Characteristic	Statistics	*N*
Age (years), mean ± SD	26.2 ± 0.4	100
Height (cm), mean ± SD	178.3 ± 8.0	100
Weight (kg), mean ± SD	76.0 ± 13.1	100
BMI (kg/m^2^), mean ± SD	23.9 ± 3.8	100
Female sex, % (n)	47.0 (47)	100
Dutch parental nationality, % (n)	98.0% (96)	98
Highest parental education		100
Low/intermediate, % (n)	38.0 (38)	
High, % (n)	62.0 (62)	
Education		94
Low/intermediate, % (n)	38.3 (36)	
High, % (n)	61.7 (58)	
Active cigarette smoker, % (n)	10.6 (10)	94
Recent cold/respiratory infection^a^	22.0 (22)	100
Current asthma^b^, % (n)	8.5 (8)	94

^a^Recent cold or respiratory infection during the 3 weeks preceding the lung function measurement. ^b^Current asthma Two out of three: asthma ever diagnosed, wheeze in past 12 months, asthma medication prescribed in past 12 months. Participant characteristics including education, asthma, smoking status, and parental nationality were missing for some participants. Missing education, asthma, or smoking data (*n* = 6) and missing parental nationality (*n* = 2) were assigned the most common response for the statistical analysis

**Table 2:** Lung function and immune activation markers

Outcome	Mean ± SD
**Lung Function**	
FEV_1_ (mL)	4,200 ± 841
FVC (mL)	5,164 ± 1,035
FEV_1_ (% of predicted)^a^	96.3 ± 10.4
FVC (% of predicted)^a^	99.0 ± 9.1
**Immune Markers (MFI)**	
Neutrophil CD11b	234,103 ± 148,580
Neutrophil CD62L	555,276 ± 109,068
Neutrophil CD10	24,752 ± 15,516
Eosinophil CD11b	44,722 ± 7,993
Non-classical monocyte CD11b	37,229 ± 13,920
Intermediate monocyte CD11b	152,036 ± 33,263
Classical monocyte CD11b	234,103 ± 148,580

Note. MFI, Median Fluorescence Intensity. ^a^Calculated with the Global Lung Initiative reference equations [26]

In analytical chemistry, the limit of detection (LOD) is typically calculated as three times the standard deviation of the average long-term procedural blank. However, in our dataset, the presence of outliers and the sporadic nature of contamination events diminished the robustness of the standard deviation as a measure of variability. To address this, we chose to define the LOD as two times the interquartile range (IQR) of the average long-term procedural blank (Table 3). The IQR is less influenced by extreme values and provides a more reliable estimate of typical variation under these conditions. This approach also offers a practical balance between bias and precision, minimizing the potential for inflated imputation rates of non-detects that could result from a higher, less representative threshold. The results were blank corrected by subtracting the median of the blank value of each polymer from the amount measured. For the statistical analysis, we considered certain polymers individually based on the number of samples measured above the LOD, as well as calculated total MNPs as the sum of all polymers measured above the LOD. For polymers detected above the LOD in more than 40% of samples, concentrations were treated as continuous variables; while those detected in 20–40% of samples were treated as binary variables (detect/non-detect). We calculated the total MNP concentration for each participant by summing concentrations above the LOD of all polymers and then imputed concentrations below the LOD. Polymers detected in < 20% of samples were only included as part of the total MNP concentration. For continuous variables, including total MNPs, values below the LOD were imputed assuming a lognormal distribution, which was chosen based on the right skewed nature of the exposure distributions. We fit an interval-censored regression model to estimate the mean and standard deviation of the uncensored distribution [27]. To handle missing values, 100 imputations were performed by drawing random numbers from the estimated lognormal distribution for each observation, conditional on the censored interval (i.e., below the LOD). Imputations were perfomed conditional on all outcomes and covariates to preserve their relationships and minimize bias in subsequent regression analyses [28]. Outcomes were used soley as predictors and were not imputed. Participants with polymer outliers, defined as values outside ± 3 standard deviations from the mean, were excluded from the analysis.

**Table 3:** MNP characteristics

Polymer	LOD (ng/mL)	Samples Above LOD *n* (%)	Median (IQR) (ng/mL)
PA6.6	5.1	5 (5.0)	< LOD
PMMA	10.9	11 (11.0)	< LOD
PS	30.1	22 (22.0)	< LOD
PP	6.7	26 (26.0)	< LOD
PE	52.3	36 (36.0)	< LOD
PET	59.5	41 (41.0)	51.2 (70.3)
PVC	62.0	99 (99.0)	266.4 (103.7)
Total	-	100 (100.0)	427.5 (160.2)

Note. LOD, limit of detection; IQR, interquartile range. The median of Total, PVC and PET are reported after imputation.

### Immune assessment

Innate immune cell activation markers were measured with three distinct antibody panels targeting neutrophils, eosinophils and monocytes. Blood was collected in a 4mL sodium heparin vacutainer (Vacuette; Greiner Bio-One) and subsequently measured by flow cytometry (AQUIOS CL, Beckman Coulter) following the ‘load & go’ procedure [29]. Measurements were carried out within one hour of venipuncture to prevent ex vivo activation of the immune cells. The antibody panels used to distinguish immune cell subsets were described in previous publications [30, 31] and are included in the supplement. From these antibody panels, immune activation was assessed by expression of CD11b, CD62L, and CD10 for neutrophils, CD11b for classical, non-classical and intermediate monocytes and CD11b for eosinophils. All immune activation markers were selected a priori. The flow cytometry data were analyzed using Cytobank (Beckman Coulter, www.cytobank.org) a web-based platform which uses the FlowSOM algorithm as a gating strategy to automatically cluster cells into distinct subsets based on forward and side scatter, as well as cell marker expression (Figure S2, S3) [30, 31]. Immune cell markers were quantified as median fluorescence intensity (MFI).

### Spirometry

Lung function, including FEV_1_ and FVC, was measured by spirometry during the same study visit when the blood sample was collected, as previously described [32]. In brief, lung function was measured by experienced technicians with EasyOne spirometers (ndd Medical Technologies Inc, Zurich, Switzerland) following the recommendations of the American Thoracic Society (ATS)/European Respiratory Society (ERS) [33]. All flow volume curves were reviewed by highly experienced lung function analysts. In the current analysis, we included lung function measurements that fulfilled the ATS/ERS criteria [33]. Measurements that did not meet these criteria (i.e., the difference between the largest and next largest values for FEV_1_ and FVC ⩽150 mL) were included if they were obtained from technically acceptable flow volume curves with the two largest FEV_1_ and FVC values within 200 mL, as in our previous analysis [32].

### Covariates

We included as potential confounders age, sex, height and weight at the time of the blood collection, recent respiratory infections (during the 3 weeks before study visit, yes/no), high education (higher vocational education or university, yes/no), high parental education (at least one parent with higher vocational education or university, yes/no), and active smoking by the participant (> 1x/week, yes/no) based on directed acyclic graphs (Figure S4) [34]. Current asthma was defined as meeting at least two of the following criteria: (1) a previous asthma diagnosis, (2) wheezing in the past 12 months, or (3) a prescription for asthma medication in the past 12 months. Participant education, asthma, and smoking status were obtained from questionnaires completed at age 23, and participants with missing covariate data (*n* = 6) were assigned the most common response.

### Statistical analysis

The internal MNP concentrations in the study population were reported using mass concentration calculated per polymer, with the concentrations of seven polymers summed to represent total MNP exposure. Splines were used to confirm linearity before proceeding with linear regression (Figure S5 and S6). We used multivariable linear regression to examine the associations of exposure to total MNPs or individual polymers (independent variables) with absolute lung function values (in mL) and immune marker MFI (dependent variables). Participants missing specific immune markers were excluded from that analysis only. Associations with continuous exposures were presented for an IQR increase in exposure. Associations with lung function are presented for minimally (age, height, weight and sex) and fully adjusted (all potential confounders described above) models. In the analysis of immune marker outcomes (1) crude associations were presented as is typical for flow cytometry data, and (2) BMI was included instead of height and weight due to the association between chronic inflammation and obesity [35]. The fully adjusted model is considered the main model for all analyses. In sensitivity analyses, we assessed (1) the impact of asthmatic participants on our findings by restricting the analysis to participants without current asthma (*n* = 86), the number of asthmatics (*n* = 8) is too small to perform a separate analysis; (2) the impact of imputing the most common response for missing covariates by excluding participants with missing covariates (i.e., complete case analysis, *n* = 6); (3) the sensitivity of associations with total MNPs to PVC (due to possible matrix interfence with PVC [36] by excluding PVC from total MNPs; and (4) the implementation of the analytical chemistry preferred LOD, calculated as three times the standard deviation of the average long-term procedural blanks or as three times the standard deviation of the per-batch procedural blanks. All statistical analyses were performed using R version 4.4.1 (The R Foundation for Statistical Computing, Vienna, Austria).

## Results

### Population characteristics

Characteristics of the study population are presented in Table 1. Participants were on average 26.2 years old, with a range between 25.6 and 27.1 years old, and 47% were female. Most participants were non-smokers (89.4%) and without current asthma (91.5%). The average percent predicted FEV_1_ and FVC were 96.3% and 99.0%, respectively (Table 2). Compared to the initial PIAMA cohort, participants of the current study were more likely to have parents with high education (Table S1).

### MNP exposure

**Fig. 1:**
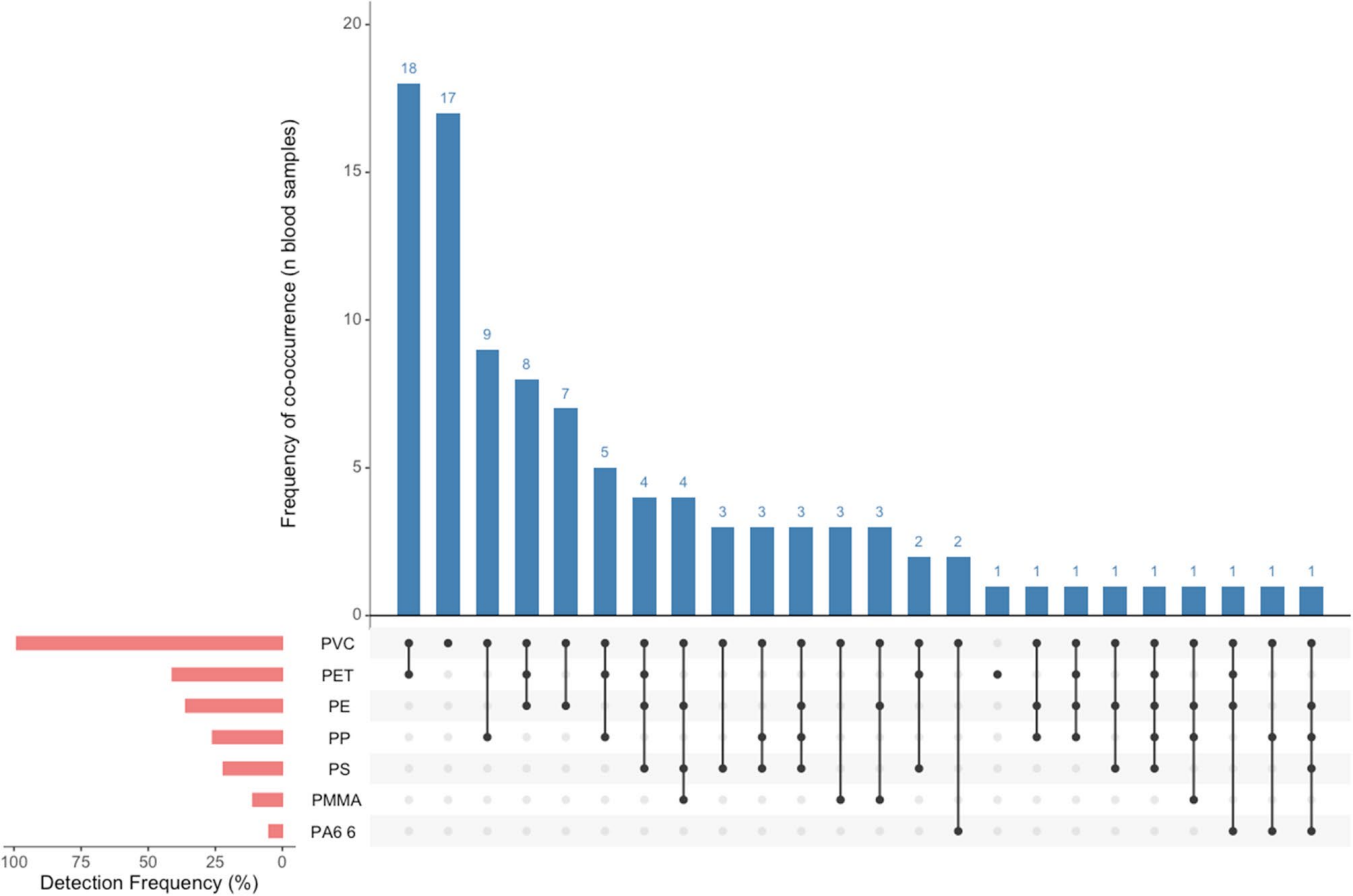
Frequency of Co-occurrent MNP Polymers

All target polymers were detected, with at least one polymer detected above the LOD in each participant (Fig. 1; Table 3). The type of polymers present per participant ranged from 1 to 5, with most participants having 2 polymers present (*n* = 42). Patterns of polymer types detected, and concentrations of individual polymers varied among participants (Fig. 1). PVC was the most frequently detected polymer (> LOD values in 99% of all participants), followed by PET (41%) and PE (36%). These three polymers were also measured at the highest concentrations. The median (interquartile range) concentration of the total MNPs measured above the LOD was 372.3 (195.6) ng/mL and the total MNP concentration ranged from 109.4 ng/mL – 784.9 ng/mL. When we imputed analyte values < LOD, the median concentration of total MNPs measured was 427.5 (160.2) ng/mL and the total MNP concentration ranged from 201.4 ng/mL – 879.7 ng/mL, across 100 imputations (Figure S7). Imputed total MNP concentrations were strongly correlated with imputed PVC (*r* = 0.73), and weakly correlated with imputed PET (*r* = 0.29) (Figure S8).

### MNPs and innate immune cell activation

**Fig. 2:**
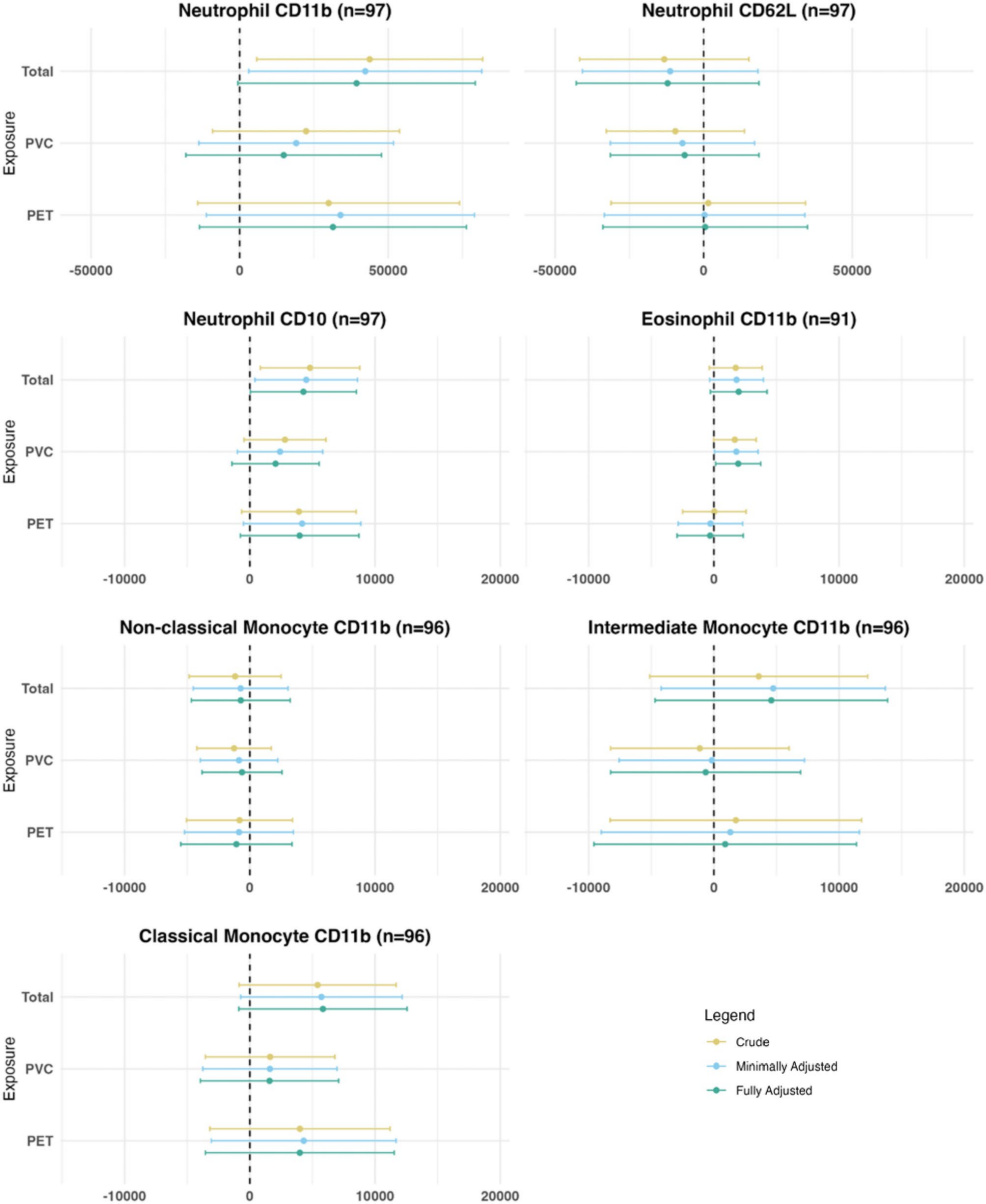
Association between MNPs and Immune Markers presented as mean difference (95% confidence interval) for an IQR increase in MNP exposure. Effect estimates and 95% confidence intervals for continuous MNP exposure variables. Associations are presented for interquartile range (IQR) increase in exposure. Crude model (yellow). Minimally adjusted model (blue) adjusted for age, BMI, and sex. Fully adjusted model (green) adjusted for age, BMI, sex, recent respiratory infection, smoking, participant education, highest parental education. X axis indicates the regression coefficient and 95% CI of the Median Fluorescence Intensity (MFI)

Higher total MNP mass concentration was associated with higher expression of CD10 in neutrophils [4,283 (95% CI 50 − 8,517)]. Further, higher total MNP mass concentrations tended to be associated with higher expression of CD11b in neutrophils [39,343 (95% CI -611–79,297)], and classical monocytes [5,842 (95% CI -879–12,563)]. For these associations there was trend between higher total MNPs and higher activation marker expression, with p-values of ≤ 0.1 for fully adjusted models. Additionally, a higher PVC concentration was associated with higher eosinophil expression of CD11b [1,946 (95% CI 146–3,746)]. MNP concentrations were not associated with non-classical or intermediate monocyte expression of CD11b or neutrophil expression of CD62L (Fig. 2 and Table S2). No associations were observed between binary exposures (PE, PP, PS) and immune markers (Table S3 and Figure S9). Results for the crude, minimally adjusted, and fully adjusted models were consistent.

### MNPs and lung function

**Fig. 3:**
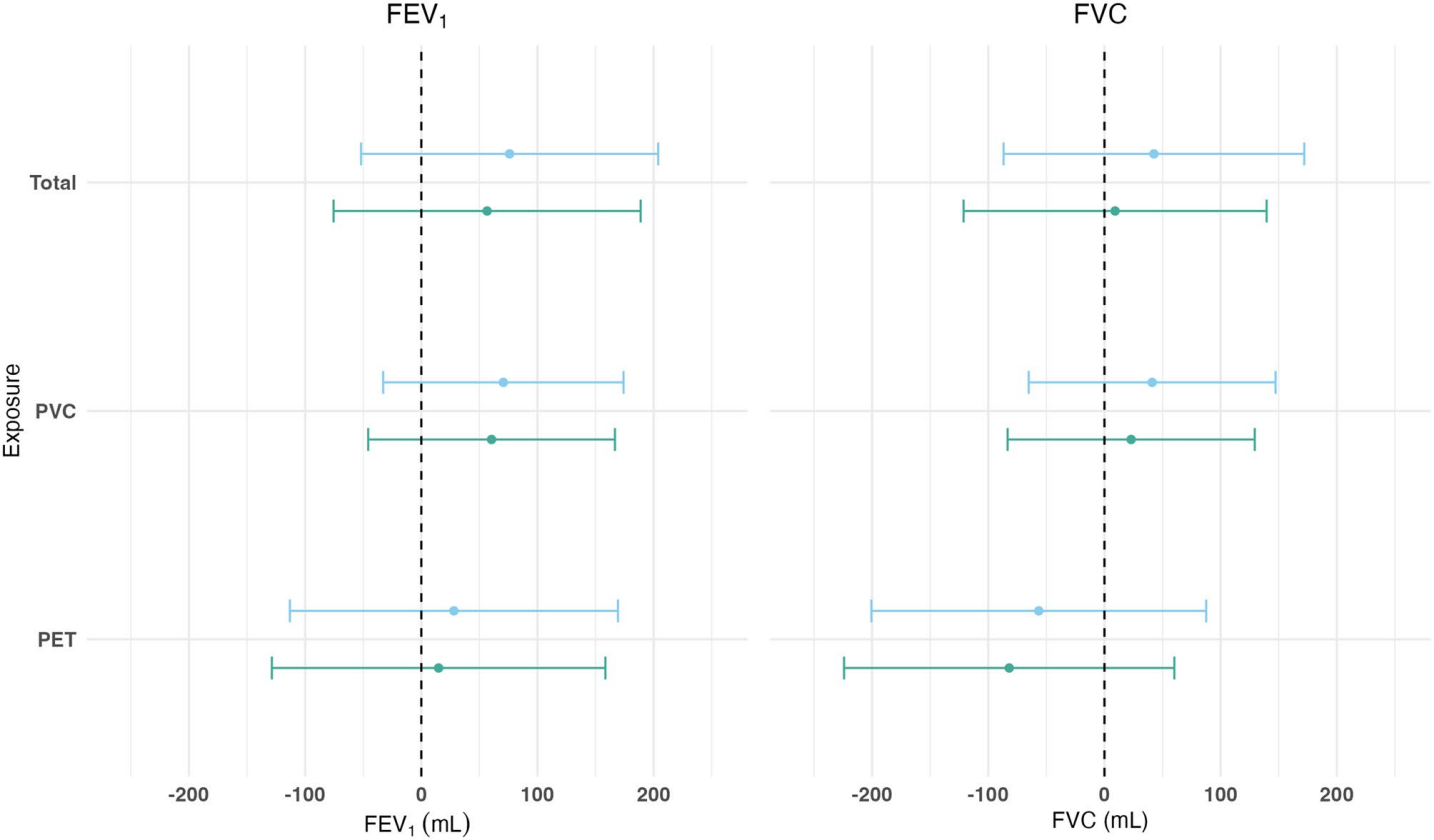
Association between MNPs and Lung Function presented as mean difference (95% confidence interval) for an IQR increase in MNP exposure (*n* = 100). Effect estimates and 95% confidence intervals for continuous MNP exposure variables. Associations are presented for interquartile range (IQR) increase in exposure. Minimally adjusted model (blue) adjusted for age, height, weight, and sex. Fully adjusted model (green) adjusted for age, height, weight, sex, recent respiratory infection, smoking, participant education, highest parental education. X axis indicates the regression coefficient and 95% CI for the exposure on FEV_1_ and FVC in mL

Both minimally and fully adjusted models showed no association between MNP exposure and FEV_1_ or FVC. This was observed for all continuous exposures (total, PVC, PET) (Fig. 3 and Table S4) and binary exposures (PE, PP, PS) (Table S5 and Figure S10).

### Sensitivity analysis

The results remained consistent with the main analysis when we restricted the analysis to participants with complete covariate data (*n* = 94) and to those without asthma (*n* = 86) (Figure S11, S12), and when we excluded PVC from total MNPs (Figure S13, S14). When we implemented the analytical chemistry preferred LOD calculated either from long-tern procedural blanks (three times the standard deviation of the average long-term procedural blank) or from per-batch procedural blanks (three times the standard deviation per batch), results were broadly in line with what we observed in the main analysis (two times the IQR of the average long-term procedural blank). Using three times the standard deviation of long-term procedural blanks, fewer measurements were above the LOD, with point estimates similar to the main analysis but confidence intervals considerably wider (Table S6 and Figure S13, S14). LODs based on per-batch blanks varied notably between batches; overall detection rates were generally consistent with the main analysis, except for PE, which showed a higher detection rate of 69% versus 36%. Point estimates remained comparable, although confidence intervals were slightly wider (Table S7, Figures S13 – S18).

## Discussion

In this cross-sectional study, we observed indications of associations between exposure to MNPs and immune marker expression in Dutch young adults. There were no associations between exposure to MNPs and lung function. The median MNP concentration in blood was 427.5 ng/mL (IQR 160.4), which is within one order of magnitude of previous studies, reporting mean concentrations of 1,600 ng/mL [6] and 268 ng/mL [7]. Of the six published studies which have measured MNPs in blood [6, 7, 37–40], only two utilized Py-GC-MS [6, 7] enabling direct comparison with the current study. 

In the current study, the predominant polymers identified, in order of decreasing abundance, were PVC, PET, and PE, differing from prior studies that identified PET, PS, and PE [6]; PE, PVC, and PET [7]; and PE, ethylene propylene diene monomer, and ethylene–vinyl acetate/ethylene vinyl alcohol [40]. The relatively small sample sizes in previous studies - analyzing 22 [6], 68 [7], and 20 [40] blood samples - along with the selection of different indicator compounds for the polymers and methods (pyrolysis vs. spectroscopy), contribute to significant variability in the findings and restricts the generalizability of MNP exposure assessment. In this study, PVC detected polymer in 99% of samples, while the second most common, PET, was detected in only 41% of samples. Given that PVC is the third most widely produced polymer globally and is widely used in food contact applications [41] and consumer products, its detection in blood is plausible. As reported by Rauert et al. [33], matrix interferences caused by inadequate sample preparation make quantifying PVC challenging resulting in high false positive rates [36, 42]. However the analytical method employed was designed for effective matrix removal as demonstrated by high recovery rates indicating that the digestion procedure successfully eliminated matrix components without compromising the integrity of the analytes. Recovery experiments showed a PVC recovery rate of 82% with a relative standard deviation of 28%, which does not suggest a high false positive rate. In addition, all polymer markers used for quantification were selected to minimize interferences and matrix effects, in particular 1,2-hydrohydronaphthalene (for PVC) and 1-eicosene (for PE) [43]. Nevertheless, we focused mostly on associations with total MNPs in the biological and health analyses. To further address this we performed a sensitivity analysis excluding PVC from total MNPs, and the results were consistent with the main analysis.

In analytical chemistry, the LOD is commonly defined as three times the standard deviation (3xSD) of the long-term procedural blank. In our dataset, however, this approach resulted in inflated LODs because the overall standard deviation was disproportionately influenced by a small number of batch-specific blank outliers. These isolated high values did not reflect typical analytical performance but nonetheless increased the LOD, thereby reducing the number of measurements above the detection threshold and increasing reliance on imputation for left-censored data. We also evaluated an alternative approach using 3xSD calculated separately for each anlytcial batch. While this reduces the influence of between-batch variability, the small number of blanks per batch (*n* = 3) made the batch-specific estimates more uncertatin and led to variable LODs across batches. To address these limitations, we adopted a non-parametric definition of the LOD based on two times the IQR (2xIQR) of the long-term procedural blanks [27]. This method is well suited for skewed or non-normal blank distributions, is robust to extreme values, and prevents undue inflation of the LOD caused by a few high blank measurements. Using 2×IQR produced detection rates comparable to the batch-specific 3 × SD approach and higher detection rates than the long-term 3 × SD approach, while reducing noise from excessive imputation below the LOD and minimizing measurement error in subsequent analyses. 

The results of our sensitivity analysis, using a LOD based on 3xSD of the long-term procedural blank, revealed high uncertainty, as indicated by wide confidence intervals. For PET and the binary exposures (PE, PP, PS), this approach could not be applied due to an insufficient number of observations above the LOD. For PVC, differences in LOD methodology had negligible impact, as only a single value fell below the LOD under both methods. The results of our sensitivity analysis, using a LOD based on 3xSD of the per-batch procedural blanks, were generally similar to those of the main analysis. Although confidence intervals were somewhat wider under the 3×SD approach, reflecting greater batch-to-batch variability, the overall conclusions remained unchanged. This indicates that both LOD definitions yield comparable results, while the 2×IQR method provides slightly greater statistical stability. Given that PE was detected above the LOD in more than 40% of samples under this alternative approach, we additionally explored potential associations with PE; however, no statistically significant associations were observed for PE or any other polymer. Given that PE was detected above the LOD in more than 40% of samples under this alternative approach, we additionally examined potential associations with PE. However, no statistically significant associations were observed for PE or any other polymer, except for PS, for which binary exposure (presence vs. absence) was associated with higher eosinophil CD11b expression. 

CD11b, also known as Integrin Alpha M (ITGAM), is a critical cell surface receptor involved in cell migration, adhesion, and transmigration over endothelium [44]. It is expressed on the surface of various immune cells including neutrophils, eosinophils, and monocytes. Upregulation of CD11b indicates immune activation and is critical for an effective immune response [45]. However, higher expression of CD11b is a consequence of immune activation, and chronic immune activation is associated with health conditions including frailty, chronic obstructive pulmonary disease, and inflammatory bowel disease [46–49]. CD10 is a membrane-bound enzyme that cleaves peptide bonds, specifically targeting and degrading small signaling peptides [50]. The expression patterns and regulatory mechanisms of CD10 are recognized as contributors to the pathogenesis of a variety of diseases including various tumors, Alzheimer’s disease, heart failure, obesity, and type-2 diabetes [50]. Thus, the observed trends of higher expression of both CD11b and CD10 in this study may therefore carry health implications, especially if replicated in future studies. In this study, we selected a priori specific innate immune system markers of inflammation for investigation; therefore, our conclusions are confined to these markers. 

We found no associations between MNP exposure and lung function in the current study. To date, only one human study has investigated MNPs in relation to lung function, reporting that higher MP levels in BALF were correlated with lower FEV_1_ and FVC in a population with clinical indication for bronchoscopy [17]. The differences in results could be attributed to variations between the study populations, as study participants were older, with 52% classified as active smokers and 77% diagnosed with interstitial lung disease [17]. Growing evidence from mouse studies supports the hypothesis that increased exposure to MNPs impairs lung function [51, 52]. The lack of such findings in the current study may be attributable to our measurements of MNPs in blood rather than directly in the lungs, the limited power, the fact that participants are young with mainly normal lung function or to the cross-sectional design, which limits the ability to establish temporal or causal relationships.

Although the current study does not support an association between MNP exposure and lung function, further investigations are warranted. Inhalation is one of the primary proposed routes of MNP exposure [3], but it is uncertain whether blood concentrations reflect long-term exposure relevant to lung function. Nonetheless, this remains pertinent as exposure to MNPs, whether through inhalation or ingestion, may be associated to systemic inflammation, which may subsequently be associated with impaired lung function [53, 54]. We are currently unable to distinguish between the contribution of different exposure routes to systemic exposure. Further, we measure immune cell activation marker expression on circulating immune cells. However, activated immune cells may extravasate to tissue sites where they are needed and therefore may not be detectable in circulation. By examining MNP exposure cross-sectionally and assuming persistent exposure, we were unable to account for any variation in exposure or temporality. The current lack of information on the half-life of blood MNP concentrations underscores the need for studies with repeated measurements in the same individual to better understand the determinants and temporal variability in exposure. 

Notably, this study represents the largest sample size to date for measuring MNP exposure in blood and is the first to investigate its associations with immune markers of inflammation and lung function. However, our study has several limitations which must be acknowledged. First, MNP exposure assessment is an emerging and rapidly evolving field. In this study a limited set of indicator compounds was selected, with one indicator representing each polymer, which could lead to uncertainties in the exact identification and quantification of the polymer [42]. Our calculation of total MNPs assumes that 1 ng of one polymer is equivalent to 1 ng of another, despite toxicological studies indicating that certain polymers, such as PA, may be more biologically active than others [55]. Nonetheless, assessing total MNPs remains valuable, as it provides insight into overall human exposure while reducing uncertainties related to polymer identification and quantification. MNPs are a complex exposure and in this study we cannot differentiate if any association originates from the particles themselves or from associated plastic-related chemicals. Second, while we do see trends of association between MNPs and selected immune markers, the cross-sectional nature of the study limits our ability to draw causal conclusions. Third, very little is known about the confounders between MNP exposures and health outcomes; therefore, we selected the confounders a priori based on our current knowledge and insights from other small particulate exposures, such as ultrafine particle air pollution [56, 57]. Further, the study population consists of a select group of participants with normal lung function with a narrow age range, most of whom have highly educated parents who were both born in the Netherlands. This limits the generalizability of findings to more diverse populations. Finally, although this study includes more participants than previous studies, the modest sample size limits the power to detect subtle associations.

## Conclusion

To conclude, our findings suggest associations between MNP exposure and immune activation markers, but no evidence of associations between MNP exposure and lung function. These findings suggest that MNPs may be associated with health perturbations in Dutch young adults. Further research is warranted to confirm these observations in larger, more diverse populations, including both healthy and clinical populations.

## Supplementary Information

Below is the link to the electronic supplementary material.

Supplementary Material 1

## Data Availability

The datasets generated and analysed during the current study are not publicly available because they contain individual-level health information that cannot be sufficiently anonymised without compromising participant privacy. Data are available from the corresponding author upon reasonable request and subject to appropriate data-sharing agreements.

## Abbreviations

ATS: American thoracic society
BALF: Bronchoalveolar lavage fluid
ERS: European respiratory society
FEV_1_: Forced expiratory volume in 1 s
FVC: Forced vital capacity
IQR: Interquartile range
LOD: Limit of detection
MFI: Median fluorescence intensity
MNP: Micro- and nanoplastic
MP: Microplastic
NP: Nanoplastic
PA6.6: Polyamide 6.6
PE: Polyethylene
PET: Polyethylene terephthalate
PIAMA: Prevention and incidence of asthma and mite allergy
PMMA: Poly(methyl methacrylate)
PP: Polypropylene
PS: Polystyrene
PVC: Polyvinyl chloride
Py-GC-MS: Pyrolysis gas chromatography mass spectrometry
QC: Quality control
