# Supplementary material for: Micro- and nanoplastic exposure, immune cell activation, and lung function in young adults
Source: Microplast nanoplast. 2026 May 7;6(1):39. doi: 10.1186/s43591-026-00189-2 (PMC13152915; doi:10.1186/s43591-026-00189-2)
Supplement: Supplementary file 1 — Supplementary Material 1 [file 43591_2026_189_MOESM1_ESM.docx]

**Supplement**

Micro- and nanoplastic exposure, immune cell activation, and lung function in Dutch young adults

Amanda M. Durkin, Tim L. P. Skrabanja, Ulrike Gehring, Runyu Zou, Virissa Lenters, Gerard H. Koppelman, Judith M. Vonk, Nienke Vrisekoop, Roel Vermeulen

**Materials and methods**

*Study design and population*

Information on demographic factors, lifestyle, household and health characteristics has been collected by questionnaires during pregnancy, when the participating children were 3 months and 1 year old and then annually until age 8 via questionnaires completed by the parents, and at ages 11 and 14, and 16 years via separate questionnaires completed by the parents and the participants. At age 25 a separate questionnaire was completed only by the participants. Medical examinations including spirometry and anthropometry were conducted at ages 8, 12, 16, and 25 years in subgroups.

*MNP exposure assessment*

The method for quantification of MNPs in human blood was performed at the Amsterdam Institute for Environment and Health, Vrije Universiteit Amsterdam and descrived in Nardella et al., 2025. The filtration was done over a 0.7 µm pore size grade glass microfiber filter (GF/F). The analysis was carried out on a pyrolysis gas chromatography mass spectrometry (Py-GC-MS) system. The instrument consisted of a multi-shot pyrolizer fitted with an auto-shot sampler (Frontier Laboratories). Double-shot analysis was performed through initial thermal desorption to remove volatile constituents, followed by pyrolysis at 600 °C to quantify the polymer content in the samples. The pyrolysate was transferred into a GC-MS (ThermoScientific Trace 1610 GC and ISQ 7610 SQMS). The MS is equipped with electron impact ionisation and data were collected in full scan mode from *m/z* 40 to 400. Polyamide 6.6 (PA6.6) was quantified by using cyclopentanone (*m/z* 41, 55, 84) and by using a calibration curve prepared from the MOMENTUM particle mixture using 5 calibration standards and therefore the results for PA6.6 are indicative values. For the remaining polymers, the following pyrolysis products were used as quantification compounds: Methyl methacrylate (*m/z* 100, 69, 41) for poly(methyl methacrylate) (PMMA); 2,4-Dimethyl-1-heptene (*m/z* 126, 83, 70) for polypropylene (PP); 5-Hexene-1,3,5-triyltribenzene (*m/z* 91, 117, 194) for polystyrene (PS); Benzoic acid (*m/z* 122, 105, 77) for polyethylene terapthalate (PET); 1,2-dihydronaphthalene (*m/z* 115, 129, 130) for polyvinyl chloride (PVC); and 1-eicosene (*m/z* 83, 97, 111) for polyethylene (PE).

*Quality Control*

Strict quality control measures were adopted to ensure reliability of the data and prevent external contamination. Cotton laboratory gloves were used and samples handling was performed in a laminar flow cabinet in a dedicated room with restricted access. All the solvents employed were filtered over glass microfiber filters. All the equipment were pre-cleaned with filtered water and when possible heated at 500 °C in a muffle oven. Procedural blanks (n=21) were processed along with the blood samples. Polymers measured in the long term procedural blanks ranged from 0 to 425 ng/ml. As there is currently no certified reference standard for MNPs in blood, quality control (QC) samples were prepared by spiking a large volume of blood from one donor with known concentrations of PMMA, PE, PP, PS, PVC, and PET. The QC samples were used to monitor the accuracy and performance of the method and maintain the quality and the reliability of the data. Identification of the pyrolysis products was made when, (1) the analyte peaks (S/N ≥ 3) from the quantifier and qualifier ions entirely overlap in the extracted ion chromatograms, (2) the ion response ratios of each of the two qualifier to the quantifier ions, acquired in scan mode, should fall within ±30% (relative) of the ratio established using calibration standards, and (3) retention times do not differ by more than 0.1 min from the average of the standards. The recoveries of the quantitation compounds for the QCs analysed in the batch analysis (n=15) ranged from 52 to 102% and the %RSDs for 14 to 44%.

*Immune Assessment*

The neutrophil antibody panel consisted of CD16-FITC, (clone 3G8), CD11b-PE (clone Bear1), CD62L-ECD (clone DREG56), CD10-PC5 (clone ALB1), and CD64-PC7 (Clone 22). The eosinophil antibody panel consisted of CD193-FITC (clone 5E8), CD44-PE (clone A32537), CD62L-ECD (clone IM2713U), CD16-PC5 (clone A07767) and CD11b-PC7 (clone A54822). The monocyte antibody panel consisted of CD11b-FITC (clone Bear1), CD169-PE (clone D21-075), CD16-ECD (clone 3G8), CD14-PC5 (clone RMO52) and HLA-DR-PC7 (clone Immuno-357). All antibodies were derived from Beckman Coulter, the Netherlands. The gating strategy used was the FlowSom algorithm in Cytobank ((Beckman Coulter, [www.cytobank.org](http://www.cytobank.org)). Neutrophils were automatically clustered into 64 clusters and 6 metaclusters and distinguished from eosinophils based on CD16/CD62L expression. Neutrophils are CD16^high^ whereas eosinophils are CD16^low^. Monocytes were automatically clustered into 100 clusters and 8 metaclusters and could be divided into three subsets; classical (CD14^high^/CD16^low^), intermediate (CD14^high^/CD16^high^) and non-classical monocytes (CD14^dim^/CD16^high^). Eosinophils were automatically clustered into 81 clusters and 8 metaclusters and identified based on CD193^high^/DC16^low^. The antibody panels and clustering procedure is similar to the panel used in de Fraiture et al 2025 (1).

Table S1. Comparison of participant characteristics between the full PIAMA cohort (N=3,963) and the current study samples

|  | **Full PIAMA cohort** | |  | **Current study sample** | |  |
| --- | --- | --- | --- | --- | --- | --- |
| **Characteristic** | **n (%)** | **N** |  | **n (%)** | **N** |  |
| Female sex | 1909 (48) | 3963 |  | 47 (47) | 100 |  |
| Dutch parental nationality | 3327 (90) | 3688 |  | 96 (98) | 98 |  |
| Highest Parental education |  |  |  |  |  |  |
| Low/intermediate | 1904 (50) | 3812 |  | 38 (38) | 100 |  |
| High | 1908 (50) | 3812 |  | 62 (62) | 100 |  |

Table S2. Association estimates for MNPs and Immune Markers, continuous exposures

|  |  | Crude | | Minimally Adjusted | | Fully Adjusted | |
| --- | --- | --- | --- | --- | --- | --- | --- |
|  |  | Estimate (95% CI) | p-value | Estimate (95% CI) | p-value | Estimate (95% CI) | p-value |
| Neutrophils (n=96) | | | | | | | |
| CD11b | Total | 43,767 (5,733 - 81,801) | 0.03* | 42,273 (3,059 - 81,488) | 0.04* | 39,343 (-611 - 79,297) | 0.06 |
|  | PVC | 22,361 (-9,110 - 53,833) | 0.17 | 19,033 (-13,717 - 51,784) | 0.26 | 14,828 (-18,095 - 47,752) | 0.38 |
|  | PET | 29,946 (-14,128 - 74,021) | 0.19 | 33,931 (-11,184 - 79,045) | 0.14 | 31,405 (-13,513 - 76,323) | 0.17 |
| CD62L | Total | -13,265 (-41,750 - 15,220) | 0.36 | -11,285 (-40,822 - 18,251) | 0.46 | -12,147 (-42,889 - 18,595) | 0.44 |
|  | PVC | -9,535 (-32,793 - 13,723) | 0.42 | -7,147 (-31,430 - 17,137) | 0.57 | -6,413 (-31,417 - 18,592) | 0.62 |
|  | PET | 1,537 (-31,194 - 34,267) | 0.93 | 298 (-33,450 - 34,046) | 0.99 | 507 (-33,923 - 34,936) | 0.98 |
| CD10 | Total | 4,810 (838 - 8,783) | 0.02* | 4,507 (409 - 8,606) | 0.03* | 4,283 (50 - 8,517) | 0.05* |
|  | PVC | 2,809 (-463 - 6,080) | 0.10 | 2,414 (-994 - 5,823) | 0.17 | 2,056 (-1,427 - 5,539) | 0.25 |
|  | PET | 3,923 (-650 - 8,497) | 0.10 | 4,178 (-508 - 8,864) | 0.08 | 3,980 (-755 - 8,715) | 0.10 |
| Eosinophils (n=90) | | | | | | | |
| CD11b | Total | 1,746 (-366 - 3,857) | 0.11 | 1,812 (-335 - 3,959) | 0.10 | 1,981 (-283 - 4,245) | 0.09 |
|  | PVC | 1,675 (-25 - 3,375) | 0.06 | 1,794 (56 - 3,532) | 0.05 | 1,946 (146 - 3,746) | 0.04* |
|  | PET | 35 (-2,500 - 2,571) | 0.98 | -273 (-2,851 - 2,304) | 0.84 | -299 (-2,943 - 2,345) | 0.83 |
| Monocytes (n=95) | | | | | | | |
| Non-classical CD11b | Total | -1,173 (-4,849 - 2,504) | 0.53 | -734 (-4,523 - 3,054) | 0.71 | -721 (-4,667 - 3,225) | 0.72 |
|  | PVC | -1,256 (-4,229 - 1,718) | 0.41 | -863 (-3,955 - 2,229) | 0.59 | -624 (-3,818 - 2,570) | 0.70 |
|  | PET | -822 (-5,058 - 3,414) | 0.70 | -864 (-5,211 - 3,483) | 0.70 | -1,073 (-5,516 - 3,370) | 0.64 |
| Intermediate CD11b | Total | 3,583 (-5,130 - 12,295) | 0.42 | 4,742 (-4,210 - 13,693) | 0.30 | 4,589 (-4,700 - 13,879) | 0.34 |
|  | PVC | -1,124 (-8,251 - 6,003) | 0.76 | -165 (-7,567 - 7,238) | 0.97 | -655 (-8,244 - 6,934) | 0.87 |
|  | PET | 1,757 (-8,288 - 11,802) | 0.73 | 1,313 (-8,997 - 11,622) | 0.80 | 907 (-9,580 - 11,394) | 0.87 |
| Classical CD11b | Total | 5,413 (-857 - 11,684) | 0.09 | 5,720 (-729 - 12,170) | 0.09 | 5,842 (-879 - 12,563) | 0.09 |
|  | PVC | 1,619 (-3,553 - 6,790) | 0.54 | 1,605 (-3,758 - 6,968) | 0.56 | 1,576 (-3,945 - 7,096) | 0.58 |
|  | PET | 4,004 (-3,194 - 11,202) | 0.28 | 4,304 (-3,072 - 11,680) | 0.26 | 3,994 (-3,546 - 11,533) | 0.30 |

Effect estimates and 95% confidence intervals for continuous MNP exposures. Associations with continuous exposures are presented for interquartile range (IQR) increase in exposure. Minimally adjusted: adjusted for age, sex, and BMI Fully adjusted: adjusted for age, sex, BMI, high parental education, high participant education, respiratory infection during the 3 weeks preceding the lung function measurement, and smoking *indicates statistical significance (p<0.05)

Table S3 Association estimates for MNPs and Immune Markers, binary exposures

|  |  | Crude | | Minimally Adjusted | | Fully Adjusted | |
| --- | --- | --- | --- | --- | --- | --- | --- |
|  |  | Estimate (95% CI) | p-value | Estimate (95% CI) | p-value | Estimate (95% CI) | p-value |
| Neutrophils (n=96) | | | | | | | |
| CD11b | PE | 44,106 (-17,556 - 105,769) | 0.16 | 39,166 (-23,787 - 102,119) | 0.23 | 29,290 (-34,337 - 92,917) | 0.37 |
|  | PP | -29,185 (-96,889 - 38,520) | 0.40 | -25,072 (-93,561 - 43,418) | 0.47 | -15,491 (-85,428 - 54,447) | 0.67 |
|  | PS | 8,813 (-63,336 - 80,962) | 0.81 | 14,428 (-58,910 - 87,766) | 0.70 | 786 (-72,501 - 74,073) | 0.98 |
| CD62L | PE | -33,502 (-78,733 - 11,730) | 0.15 | -30,677 (-77,065 - 15,711) | 0.20 | -30,835 (-78,806 - 17,136) | 0.21 |
|  | PP | 9,190 (-40,662 - 59,042) | 0.72 | 7,058 (-43,582 - 57,699) | 0.79 | 3,039 (-49,971 - 56,050) | 0.91 |
|  | PS | -13,650 (-66,557 - 39,258) | 0.61 | -15,987 (-70,028 - 38,054) | 0.56 | -10,179 (-65,632 - 45,275) | 0.72 |
| CD10 | PE | 2,411 (-4,077 - 8,898) | 0.47 | 1,805 (-4,812 - 8,421) | 0.59 | 1,181 (-5,598 - 7,960) | 0.73 |
|  | PP | -1,084 (-8,177 - 6,009) | 0.77 | -713 (-7,884 - 6,458) | 0.85 | -306 (-7,735 - 7,124) | 0.94 |
|  | PS | 1,916 (-5,611 - 9,443) | 0.62 | 2,467 (-5,181 - 10,116) | 0.53 | 1,266 (-6,507 - 9,040) | 0.75 |
| Eosinophils (n=90) | | | | | | | |
| CD11b | PE | -1,185 (-4,634 - 2,265) | 0.50 | -1,066 (-4,546 - 2,413) | 0.55 | -1,127 (-4,746 - 2,492) | 0.54 |
|  | PP | -915 (-4,768 - 2,937) | 0.64 | -832 (-4,730 - 3,065) | 0.68 | -810 (-4,926 - 3,307) | 0.70 |
|  | PS | -1,006 (-4,988 - 2,977) | 0.62 | -529 (-4,579 - 3,522) | 0.80 | -184 (-4,386 - 4,018) | 0.93 |
| Monocytes (n=95) | | | | | | | |
| Non-classical CD11b | PE | -5,721 (-11,459 - 16) | 0.05 | -5,356 (-11,221 - 508) | 0.08 | -4,966 (-11,053 - 1,120) | 0.11 |
|  | PP | -1,301 (-7,761 - 5,158) | 0.69 | -1,466 (-8,011 - 5,079) | 0.66 | -1,423 (-8,262 - 5,416) | 0.68 |
|  | PS | -1,573 (-8,337 - 5,191) | 0.65 | -1,857 (-8,736 - 5,023) | 0.60 | -1,482 (-8,561 - 5,597) | 0.68 |
| Intermediate CD11b | PE | 1,971 (-12,010 - 15,952) | 0.78 | 3,200 (-11,047 - 17,447) | 0.66 | 2,441 (-12,213 - 17,095) | 0.74 |
|  | PP | -3,436 (-18,869 - 11,996) | 0.66 | -3,758 (-19,400 - 11,884) | 0.64 | -2,151 (-18,399 - 14,098) | 0.80 |
|  | PS | 5,025 (-11,124 - 21,175) | 0.54 | 5,200 (-11,234 - 21,634) | 0.54 | 3,200 (-13,612 - 20,012) | 0.71 |
| Classical CD11b | PE | 5,726 (-4,372 - 15,823) | 0.27 | 5,602 (-4,686 - 15,889) | 0.29 | 5,546 (-5,075 - 16,167) | 0.31 |
|  | PP | -4,520 (-15,708 - 6,668) | 0.43 | -3,895 (-15,234 - 7,444) | 0.50 | -2,570 (-14,403 - 9,262) | 0.67 |
|  | PS | -1,343 (-13,098 - 10,412) | 0.82 | -409 (-12,362 - 11,543) | 0.95 | -1,454 (-13,711 - 10,802) | 0.82 |

Effect estimates and 95% confidence intervals for each MNP exposure variable. Associations with continuous exposures are presented for interquartile range (IQR) increase in exposure. Minimally adjusted: adjusted for age, sex, and BMI Fully adjusted: adjusted for age, sex, BMI, high parental education, high participant education, respiratory infection during the 3 weeks preceding the lung function measurement, and smoking *indicates statistical significance (p<0.05)

Table S4. Association estimates for MNPs and Lung Function, continuous exposures (n=100)

|  | FEV_1_ | | | FVC | | |
| --- | --- | --- | --- | --- | --- | --- |
| Exposure | Estimate | 95% CI | p-value | Estimate | 95% CI | p-value |
| *Minimally Adjusted* |  |  |  |  |  |  |
| Total | 76.00 | (-51.87, 203.88) | 0.25 | 42.62 | (-86.80, 172.03) | 0.52 |
| PVC | 70.61 | (-32.85, 174.07) | 0.18 | 41.06 | (-65.18, 147.31) | 0.45 |
| PET | 28.05 | (-113.13, 169.23) | 0.70 | -56.50 | (-200.58, 87.59) | 0.44 |
| *Fully Adjusted* |  |  |  |  |  |  |
| Total | 56.63 | (-75.52, 188.77) | 0.40 | 9.19 | (-121.23, 139.61) | 0.89 |
| PVC | 60.46 | (-45.74, 166.65) | 0.27 | 23.00 | (-83.36, 129.36) | 0.67 |
| PET | 14.87 | (-128.66, 158.4) | 0.84 | -81.97 | (-224.11, 60.18) | 0.26 |

Effect estimates and 95% confidence intervals for continuous MNP exposures. Associations are presented for interquartile range (IQR) increase in exposure

Minimally adjusted: adjusted for age, sex, height, weight

Fully adjusted: adjusted for age, sex, height, weight, high parental education, high participant education, respiratory infection during the 3 weeks preceding the lung function measurement, and smoking

Table S5. Association estimates for binary MNPs and Lung Function, binary exposures (n=100)

|  | FEV_1_ | | | FVC | | |
| --- | --- | --- | --- | --- | --- | --- |
| Exposure | Estimate | 95% CI | p-value | Estimate | 95% CI | p-value |
| *Minimally Adjusted* |  |  |  |  |  |  |
| PP | 103.39 | (-111.10, 317.87) | 0.35 | 46.41 | (-173.28, 266.10) | 0.68 |
| PE | 10.70 | (-185.79, 207.18) | 0.92 | -20.60 | (-221.05, 179.85) | 0.84 |
| PS | -42.82 | (-270.04, 184.4) | 0.71 | -0.63 | (-232.64, 231.39) | 1.00 |
| *Fully Adjusted* |  |  |  |  |  |  |
| PP | 119.29 | (-104.78, 343.36) | 0.3 | 60.55 | (-163.56, 284.65) | 0.60 |
| PE | -20.69 | (-223.26, 181.89) | 0.84 | -59.74 | (-261.11, 141.63) | 0.56 |
| PS | -59.40 | (-290.40, 171.60) | 0.62 | -31.35 | (-261.59, 198.89) | 0.79 |

Effect estimates and 95% confidence intervals for Ω MNP exposures.

Minimally adjusted: adjusted for age, sex, height, weight

Fully adjusted: adjusted for age, sex, height, weight, high parental education, high participant education, respiratory infection during the 3 weeks preceding the lung function measurement, and smoking

Table S6. Comparison of LOD and number of samples above LOD for different LOD calculation methods

|  | **LOD: 2xIQR of average long-term procedural blank** | | **LOD: 3xSD of average long-term procedural blank** | | | **LOD: 3xSD of per batch procedural blank*** | |
| --- | --- | --- | --- | --- | --- | --- | --- |
| **Polymer** | **LOD**  **ng/ml** | **Samples Above LOD**  **n (%)** | **LOD**  **ng/ml** | **Samples Above LOD**  **n (%)** | **LOD**  **ng/ml** | | **Samples Above LOD**  **n (%)** |
| PA6.6 | 5.1 | 5 (5.0) | 8.3 | 4 (4.0) | 3.3 – 19.2 | | 4 (4.0) |
| PMMA | 10.9 | 11 (11.0) | 48.8 | 3 (3.0) | 1.3 – 133.8 | | 24 (24.0) |
| PS | 30.1 | 22 (22.0) | 52.5 | 9 (9.0) | 8.0 – 91.4 | | 27 (27.0) |
| PP | 6.7 | 26 (26.0) | 14.4 | 14 (14.0) | 2.6 – 20.9 | | 24 (24.0) |
| PE | 52.3 | 36 (36.0) | 245.0 | 1 (1.0) | 12.4 – 604.3 | | 69 (69.0) |
| PET | 59.5 | 41 (41.0) | 79.4 | 30 (30.0) | 7.8 – 125.0 | | 45 (45.0) |
| PVC | 62.0 | 99 (99.0) | 120.3 | 99 (99.0) | 18.9 – 225.9 | | 86 (86.0) |

*Range of LODs is given representing the minimum and maximum batch specific LODs. Seven batches were analyzed, each with three procedural blanks. Abbreviations: LOD, limit of detection; IQR, interquartile range; SD, standard deviation.

Table S7. LODs calculated as 3×SD of per-batch procedural blanks and corresponding numbers of samples above the LOD.

| **Batch** | **1** | **2** | **3** | **4** | **5** | **6** | **7** | **Overall** |
| --- | --- | --- | --- | --- | --- | --- | --- | --- |
| **Number of Samples** | 13 | 16 | 16 | 15 | 15 | 15 | 10 |  |
| **PA6.6** | | | | | | | | |
| LOD (ng/ml) | 9.2 | 19.2 | 5.2 | 4.0 | 4.7 | 3.3 | 4.3 |  |
| Samples Above LOD, n (%) | 0 (0.0) | 0 (0.0) | 0 (0.0) | 1 (6.7) | 2 (13.3) | 1 (6.7) | 0 (0.0) | 4 (4.0) |
| **PMMA** | | | | | | | | |
| LOD (ng/ml) | 1.7 | 6.9 | 3.9 | 10.2 | 133.8 | 4.8 | 1.3 |  |
| Samples Above LOD, n (%) | 8 (61.5) | 3 (18.8) | 5 (31.2) | 2 (13.3) | 0 (0.0) | 0 (0.0) | 6 (60.0) | 24 (24.0) |
| **PS** | | | | | | | | |
| LOD (ng/ml) | 21.4 | 26.9 | 91.4 | 8.0 | 42.4 | 9.4 | 28.1 |  |
| Samples Above LOD, n (%) | 9 (69.2) | 0 (0.0) | 1 (6.2) | 10 (66.7) | 3 (20.0) | 2 (13.3) | 2 (20.0) | 27 (27.0) |
| **PP** | | | | | | | | |
| LOD (ng/ml) | 2.9 | 20.9 | 2.6 | 5.2 | 16.9 | 13.0 | 8.0 |  |
| Samples Above LOD, n (%) | 2 (15.4) | 0 (0.0) | 11 (68.8) | 6 (40.0) | 4 (26.7) | 1 (6.7) | 0 (0.0) | 24 (24.0) |
| **PE** | | | | | | | | |
| LOD (ng/ml) | 33.3 | 604.3 | 33.0 | 13.4 | 12.3 | 14.7 | 15.6 |  |
| Samples Above LOD, n (%) | 11 (84.6) | 0 (0.0) | 9 (56.2) | 14 (93.3) | 15 (100.0) | 11 (73.3) | 9 (90.0) | 69 (69.0) |
| **PET** | | | | | | | | |
| LOD (ng/ml) | 48.8 | 16.8 | 125.0 | 9.8 | 7.8 | 67.7 | 29.9 |  |
| Samples Above LOD, n (%) | 3 (23.1) | 11 (68.8) | 0 (0.0) | 10 (66.7) | 14 (93.3) | 4 (26.7) | 3 (30.0) | 45 (45.0) |
| **PVC** | | | | | | | | |
| LOD (ng/ml) | 51.9 | 225.9 | 74.8 | 37.7 | 18.9 | 25.2 | 33.7 |  |
| Samples Above LOD, n (%) | 13 (100.0) | 3 (18.8) | 15 (93.8) | 15 (100.0) | 15 (100.0) | 15 (100.0) | 10 (100.0) | 86 (86.0) |

Seven batches were analyzed, each with three procedural blanks. Note. LOD, limit of detection

Figure S1. Flow-diagram of PIAMA study population selection.


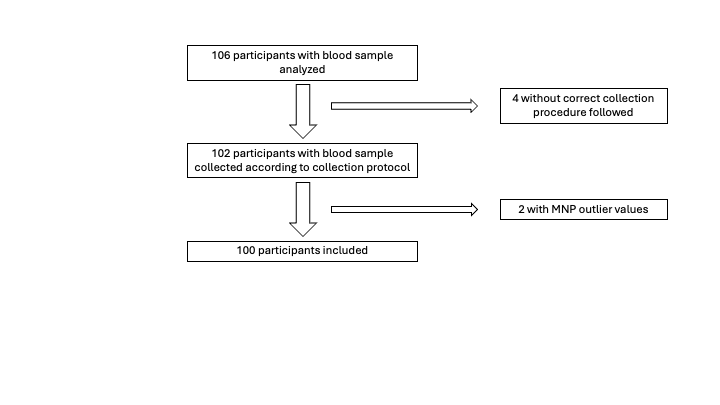


Figure S2. Representative image of the gating strategy for mature neutrophils (metacluster 4) by using the FLowSom algorithm with 64 clusters and 6 metaclusters. (A) The expression of the measured markers of mature neutrophils (MFI) for CD11b, CD10 and CD62L (B).


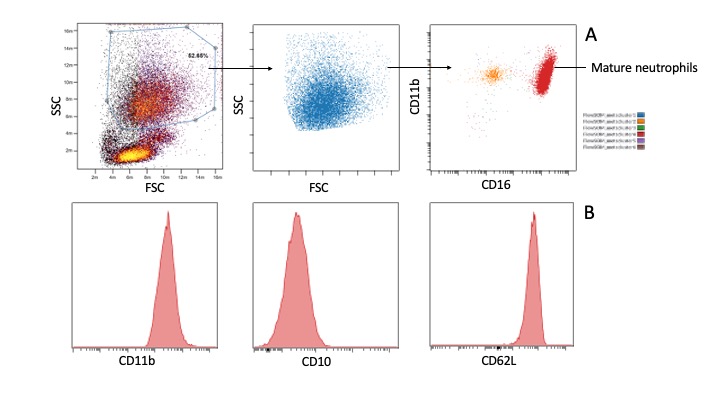


Figure S3. Representative image of the gating strategy for non-classical monocytes (metacluster 4), intermediate monocytes (metacluster 5) and classical monocytes (metacluster 8) by using the FLowSom algorithm with 100 clusters and 8 metaclusters (A) and for eosinophils with 81 clusters and 8 metaclustes (C). The expression of the measured marker CD11b (MFI) per monocyte subset (B) and for eosinophils (D).


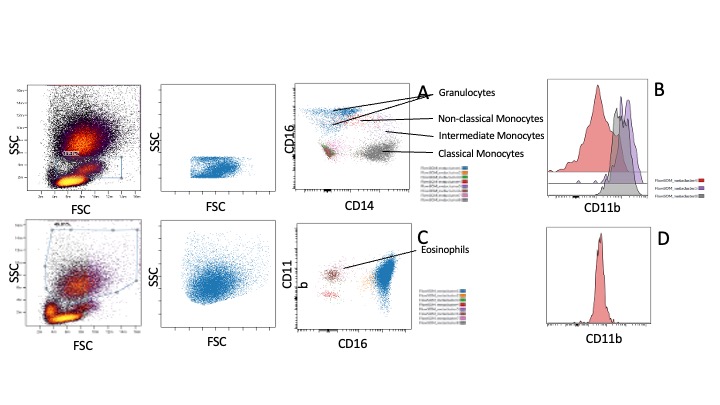


Figure S4. Direct acyclic graph depicting the relationship between exposure to MNPs, lung function and immune activation covariates


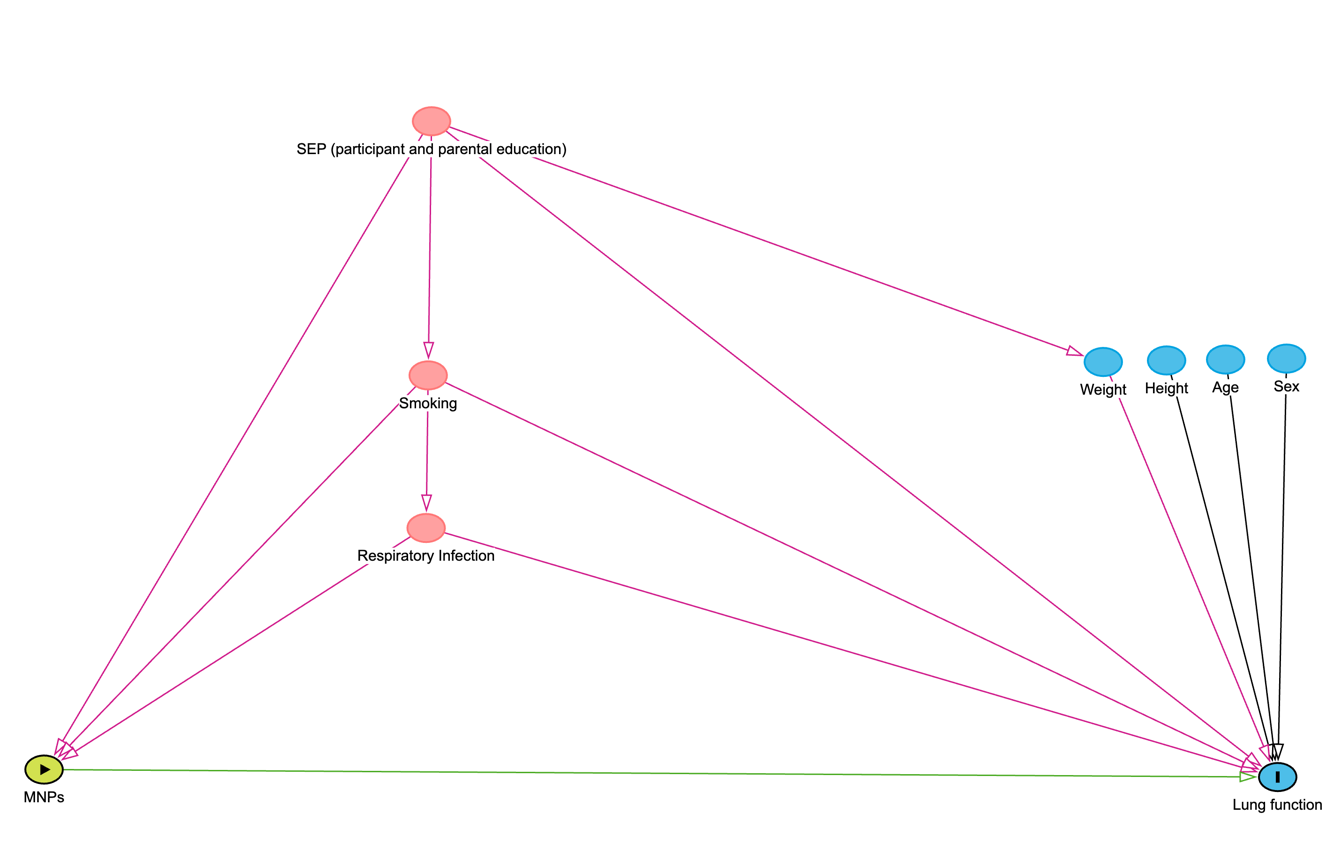

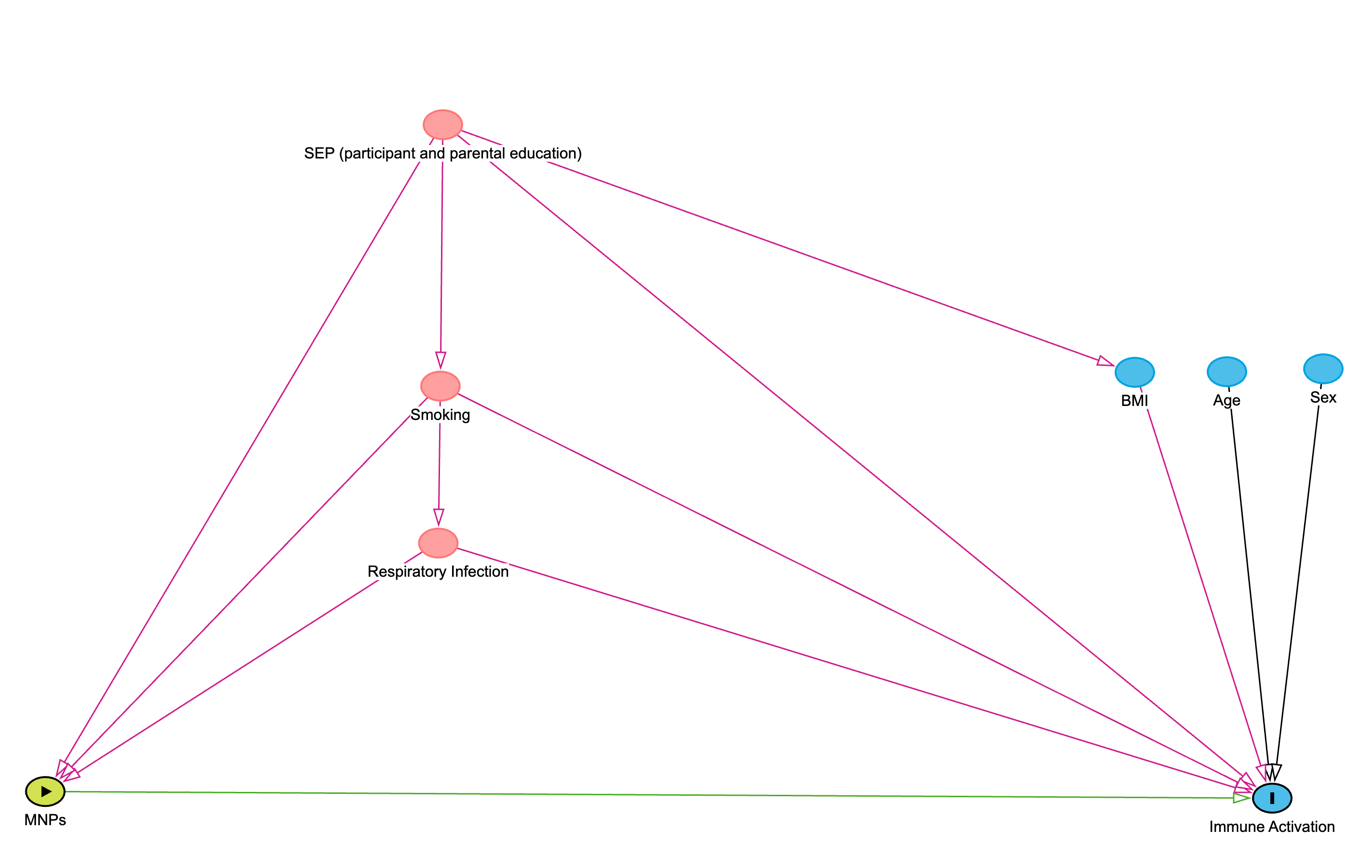


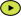
exposure,
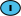
 outcome,
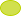
 ancestor of exposure,
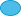
 ancestor of outcome,
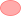
 ancestor of exposure *and* outcome,
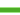
 causal path,
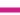
 biasing path

Note. The graph is available at [https://doi.org/10.83031/6lwex6ix](https://eur03.safelinks.protection.outlook.com/?url=https%3A%2F%2Fdoi.org%2F10.83031%2F6lwex6ix&data=05%7C02%7Ca.durkin%40uu.nl%7Cfd95d600c18a4acbd83d08de8431faa8%7Cd72758a0a4464e0fa0aa4bf95a4a10e7%7C0%7C0%7C639093547359130502%7CUnknown%7CTWFpbGZsb3d8eyJFbXB0eU1hcGkiOnRydWUsIlYiOiIwLjAuMDAwMCIsIlAiOiJXaW4zMiIsIkFOIjoiTWFpbCIsIldUIjoyfQ%3D%3D%7C0%7C%7C%7C&sdata=ayKWLFJ69vDmplYcr%2BifFHm7HbayturSNtXEbF9mzhg%3D&reserved=0) (2). Abbreviations: MNPs, Micro and nano-plastics; SEP, Socioeconomic Position, BMI, Body Mass Index.

Figure S5. Smoothed exposure-response relationships^1^ with immue activation markers

|  | | **Total** | | **PVC** | **PET** |
| --- | --- | --- | --- | --- | --- |
| Neutrophil  CD11b | 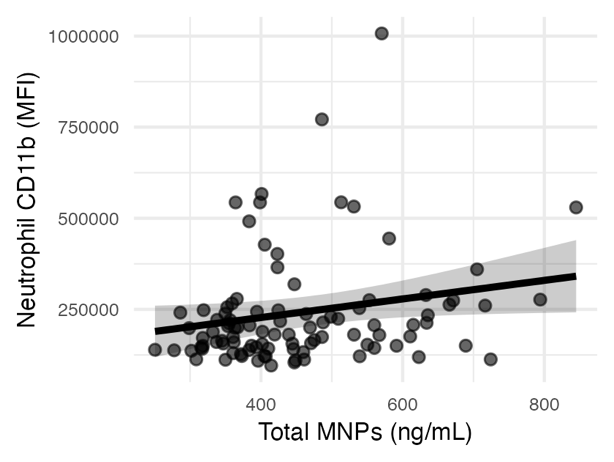 | | 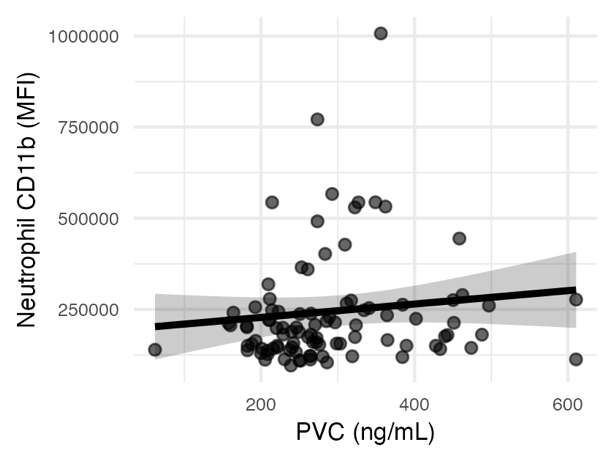 | | 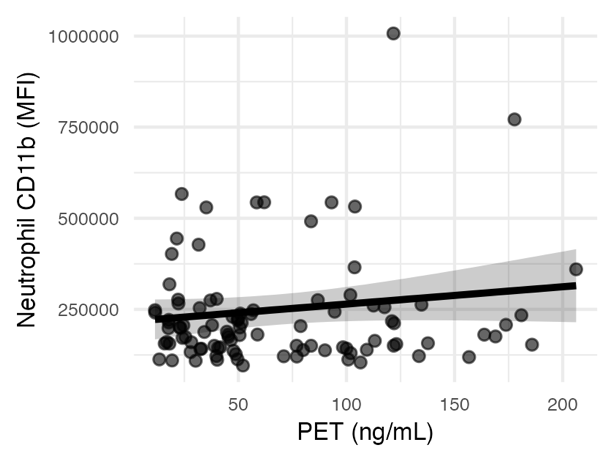 |
| Neutrophil  CD62L | 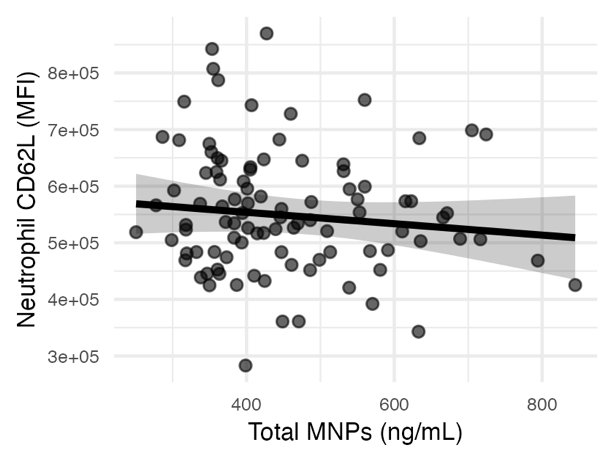 | | 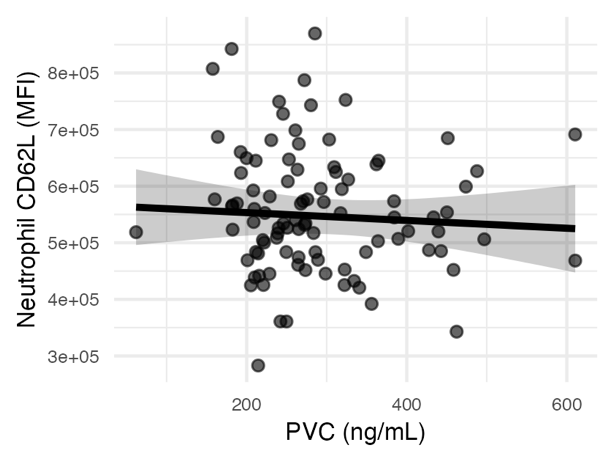 | | 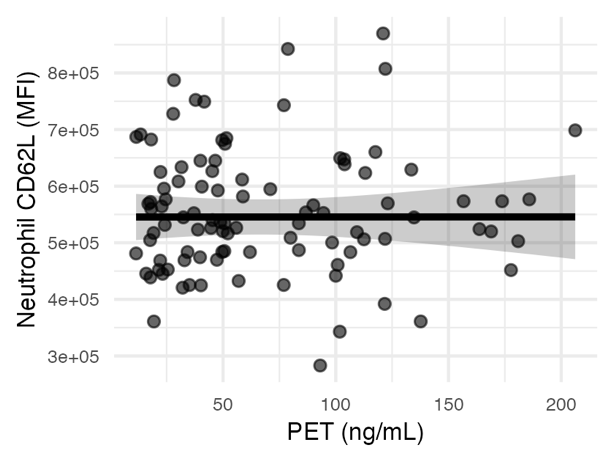 |

^1^Natural splines with 6 knots, adjusted for sex, age, and BMI.

Figure S5. (continued)

|  | **Total** | **PVC** | **PET** |
| --- | --- | --- | --- |
| Neutrophil  CD10 | 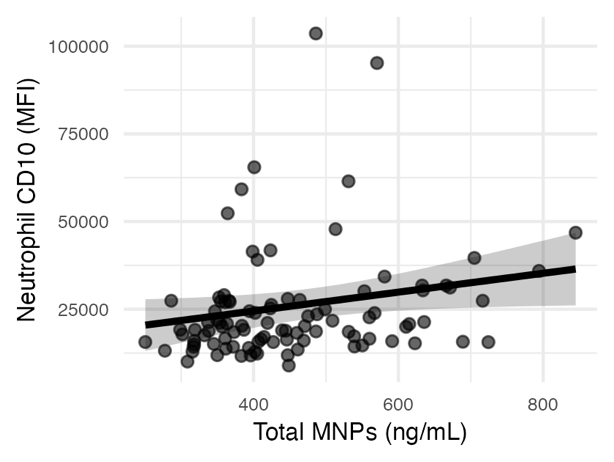 | 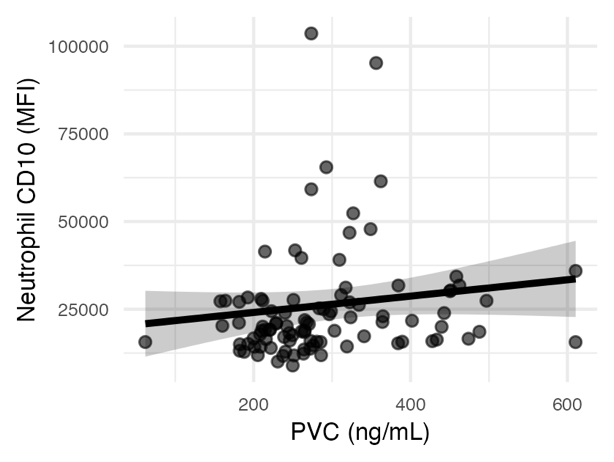 | 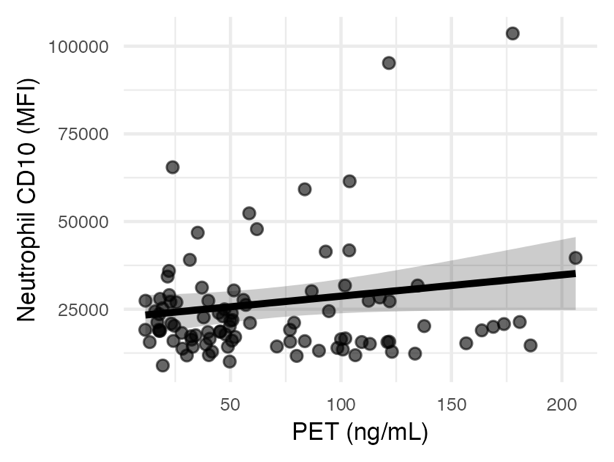 |
| Eosinophil  CD11b | 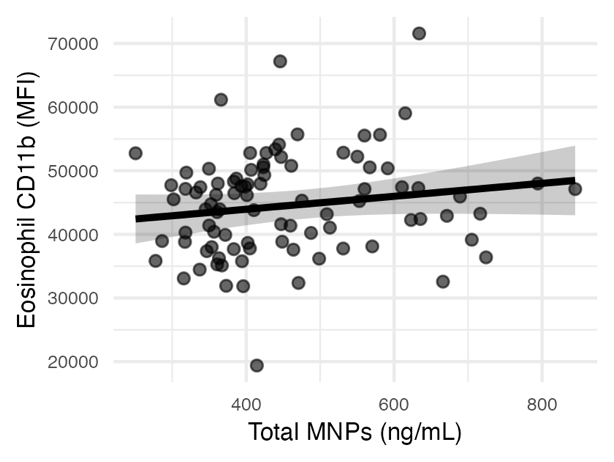 | 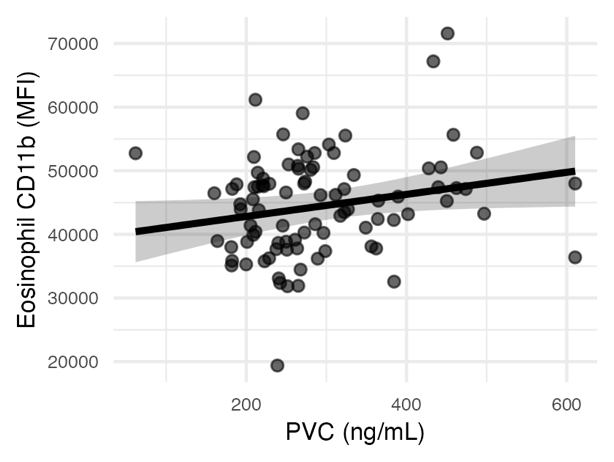 | 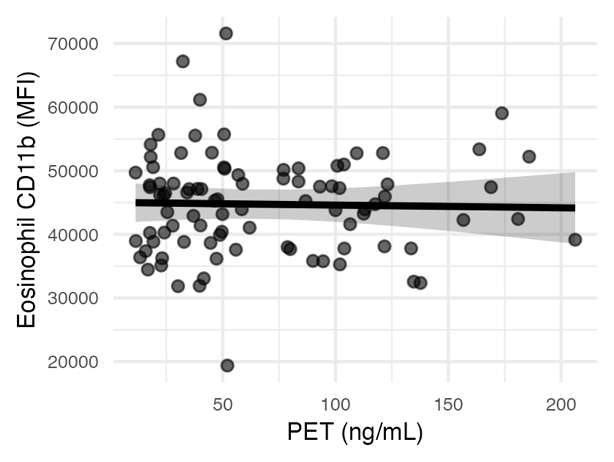 |

^1^Natural splines with 6 knots, adjusted for sex, age, and BMI.

Figure S5. (continued)

|  | **Total** | **PVC** | **PET** |
| --- | --- | --- | --- |
| Non-classical  CD11b | 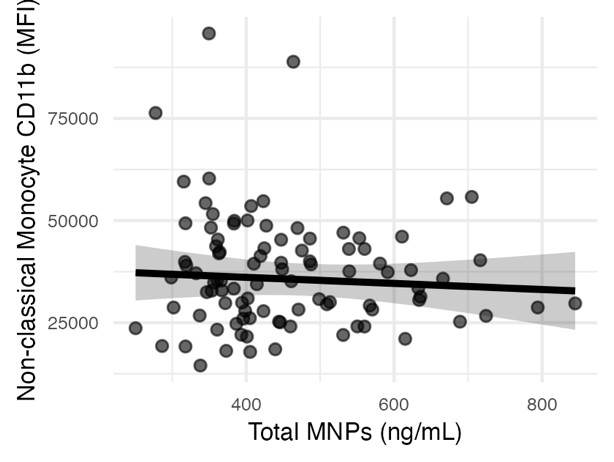 | 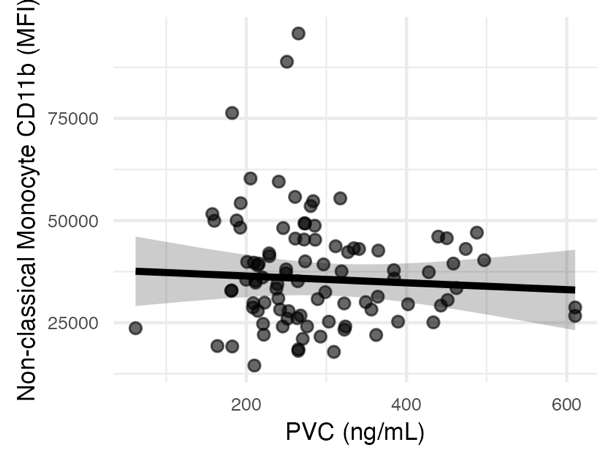 | 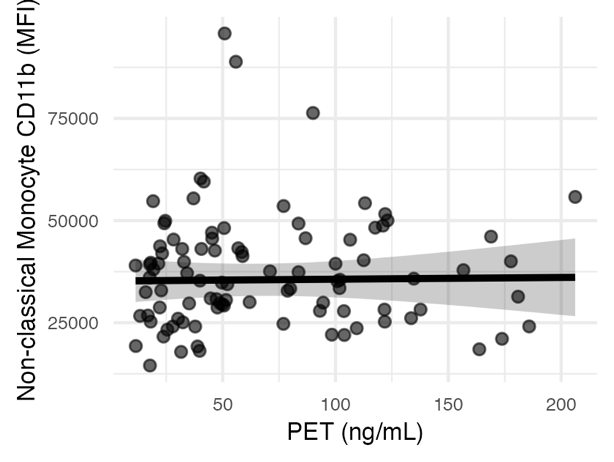 |
| Intermediate  CD11b | 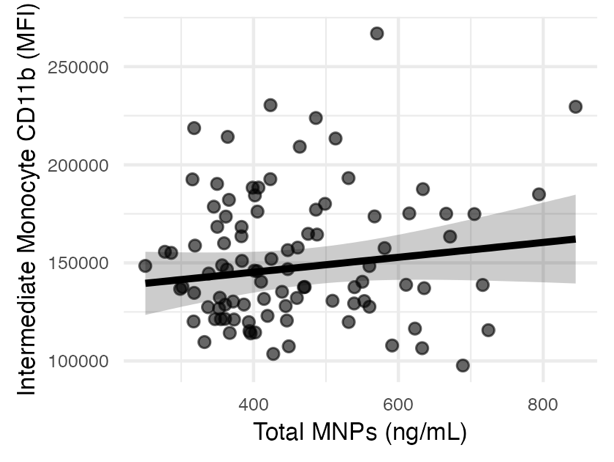 | 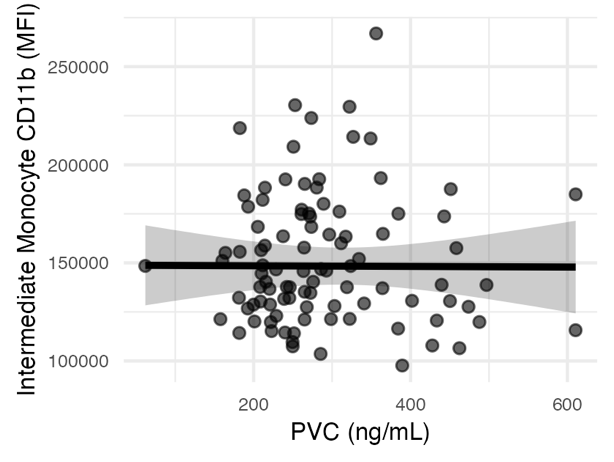 | 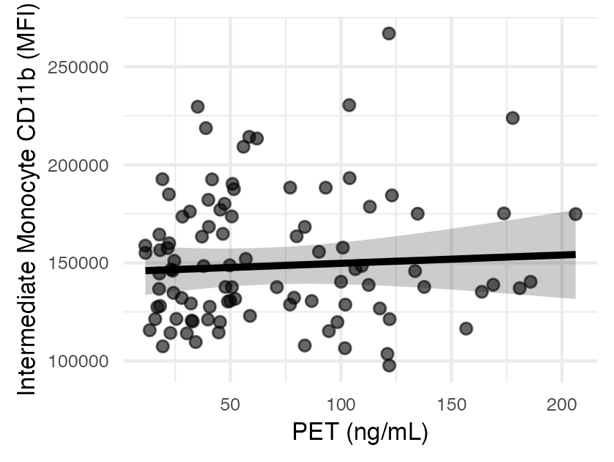 |

^1^Natural splines with 6 knots, adjusted for sex, age, and BMI.

Figure S5. (continued)

|  | **Total** | **PVC** | **PET** |
| --- | --- | --- | --- |
| Classical  CD11b | 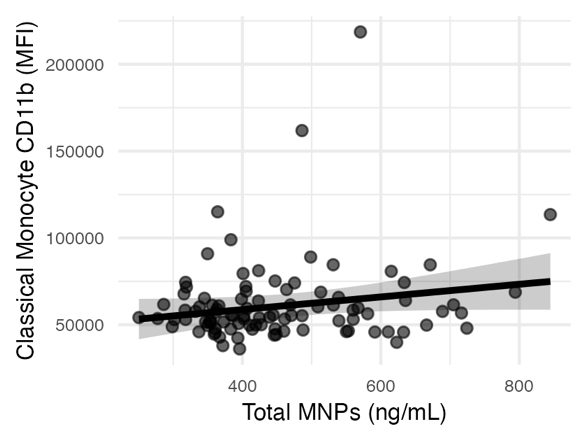 | 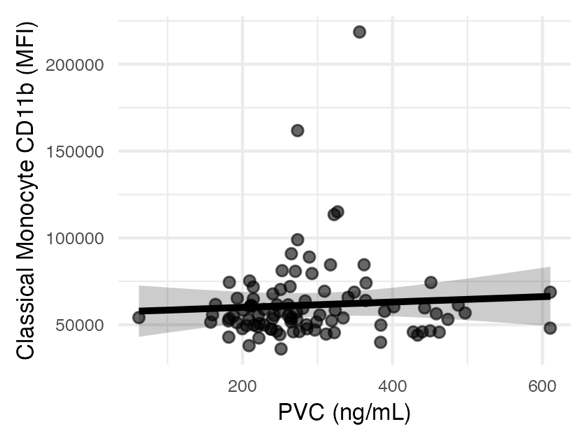 | 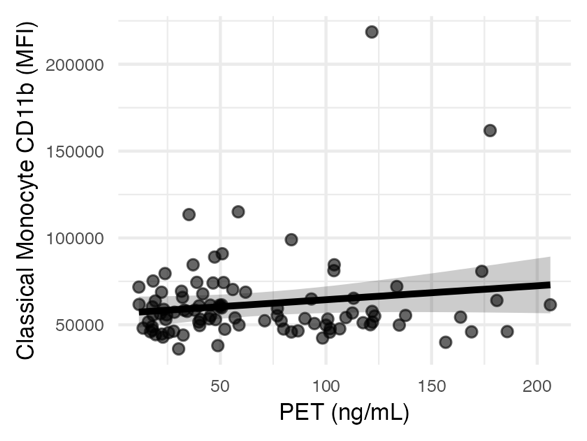 |

^1^Natural splines with 6 knots, adjusted for sex, age, and BMI.

Figure S6. Smoothed exposure-response relationships^1^ with FEV_1_ and FVC

|  | **Total** | **PVC** | **PET** |
| --- | --- | --- | --- |
| FEV_1_ | 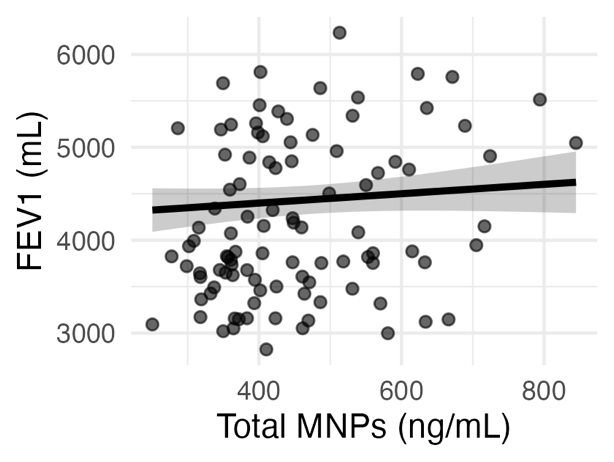 | 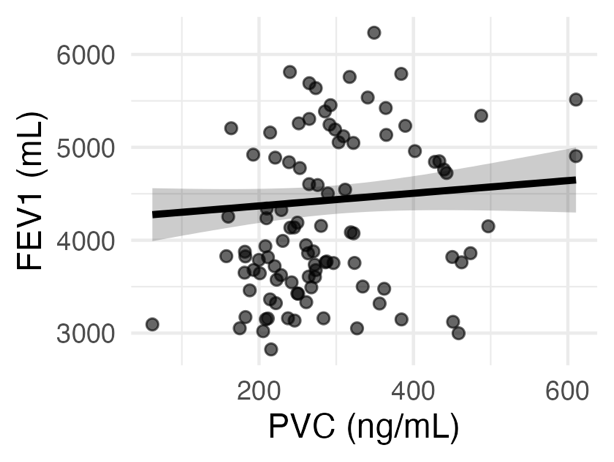 | 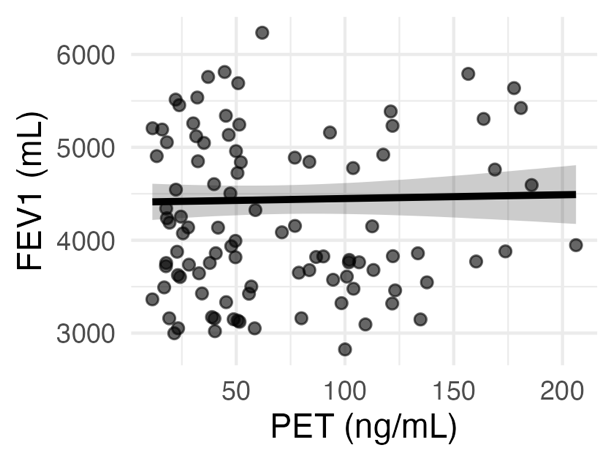 |
| FVC | 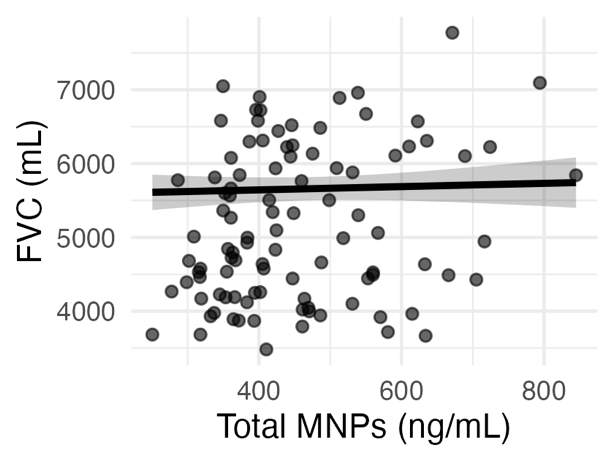 | 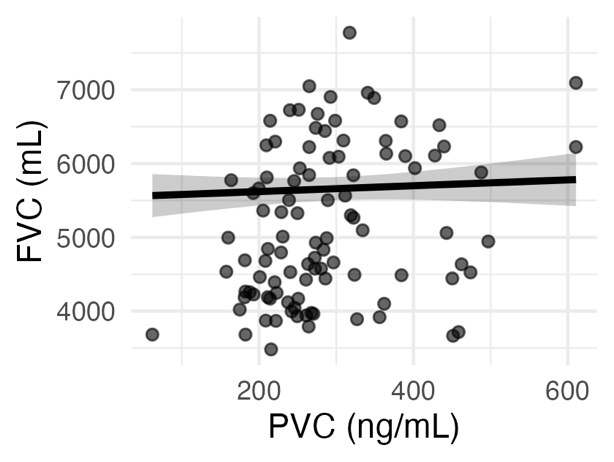 | 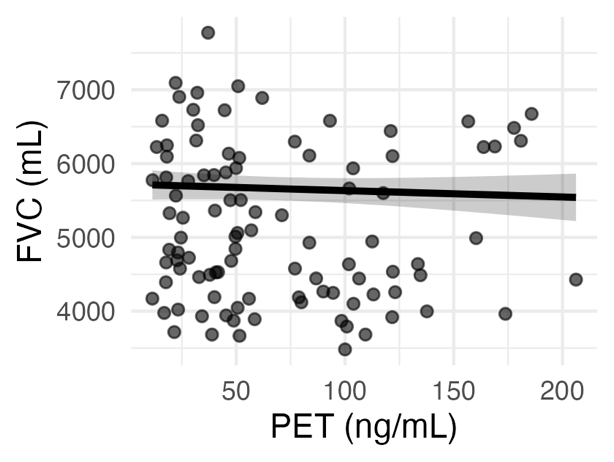 |

^1^Natural splines with 6 knots, adjusted for sex, age, height and weight

.

Figure S7. Histograms of distributions for imputed continuous MNP exposures


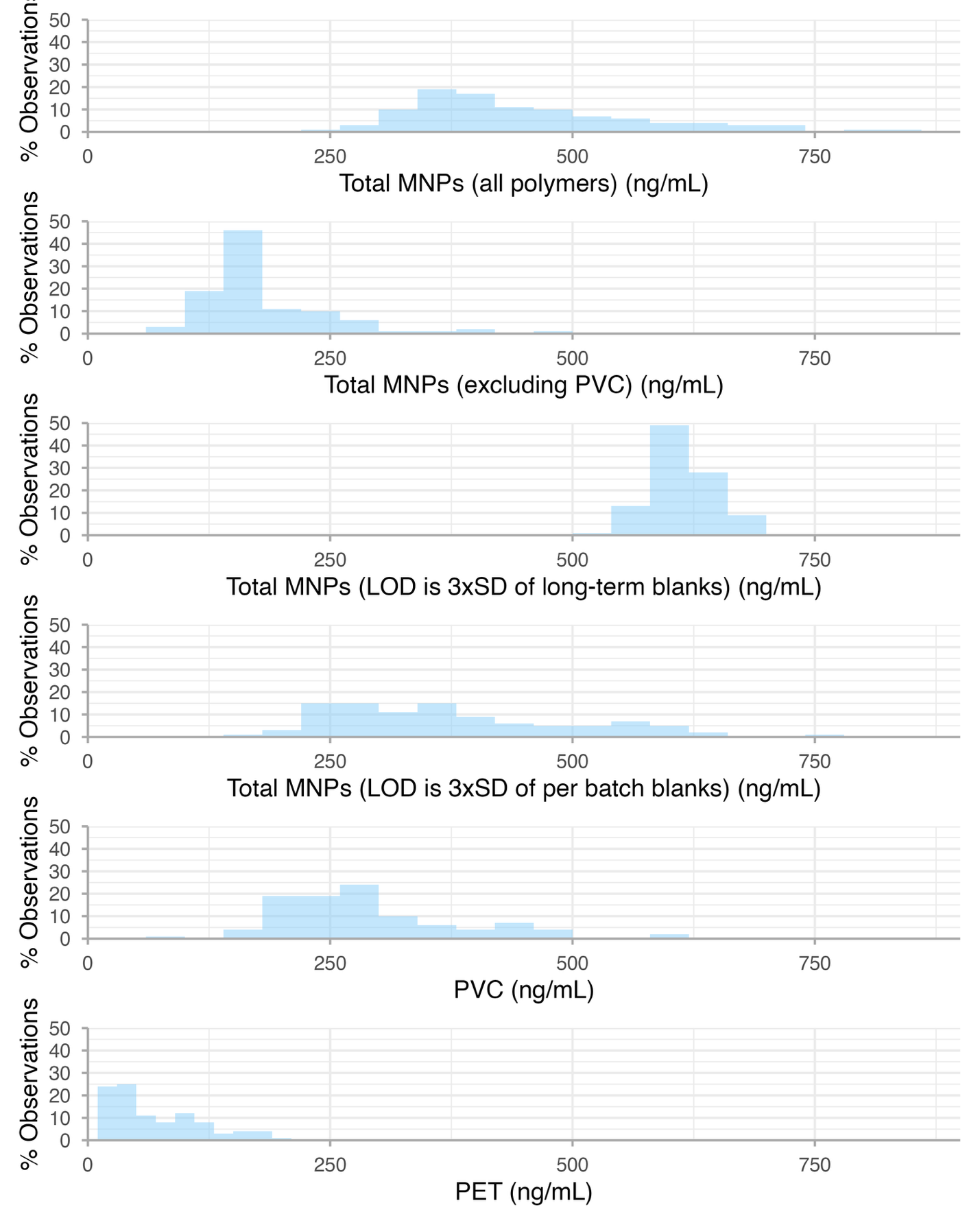


Figure S8. Spearman correlation for continuous MNP exposures


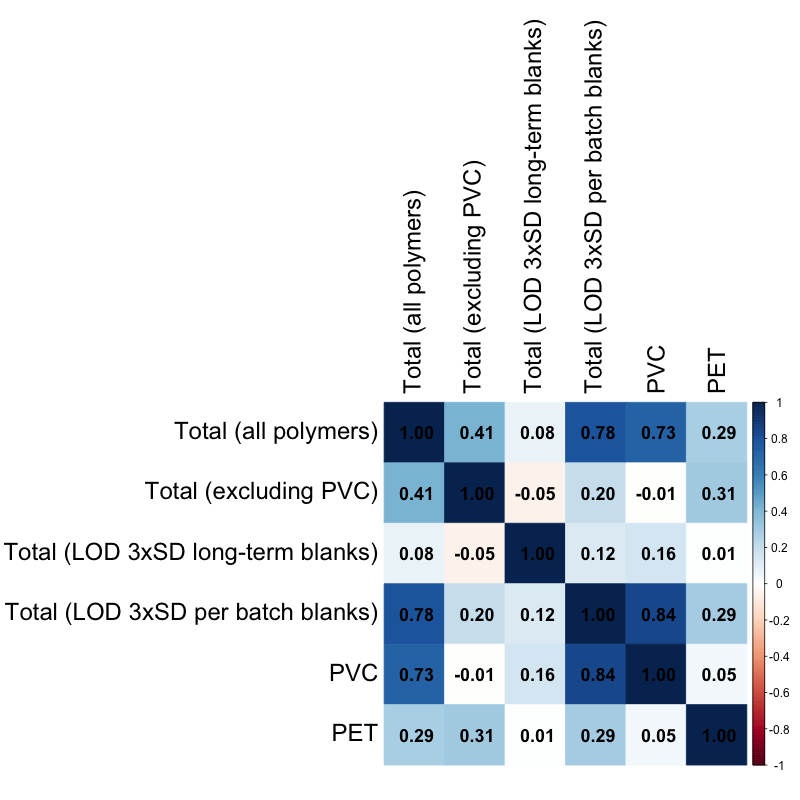


Correlation coefficients are presented for the first imputed data set

Figure S9. Association between MNPs and Immune Markers presented as mean difference (95% confidence interval) for an IQR increase in MNP exposure, binary exposures


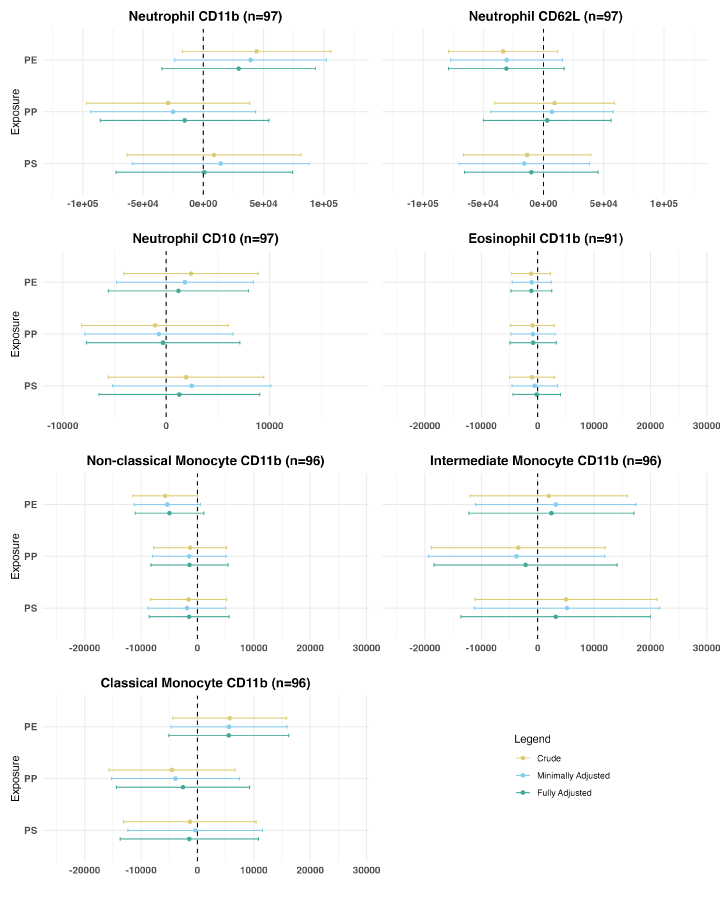


Effect estimates and 95% confidence intervals for binary MNP exposure variables. Crude model (yellow). Minimally adjusted model (blue) adjusted for age, BMI, and sex. Fully adjusted model (green) adjusted for age, BMI, sex, recent respiratory infection, smoking, participant education, highest parental education. X axis indicates the regression coefficient and 95% CI of the Median Fluorescence Intensity (MFI).

Figure S10. Association between MNPs and Lung Function presented as mean difference (95% confidence interval) for an IQR increase in MNP exposure, binary exposures (n=100)


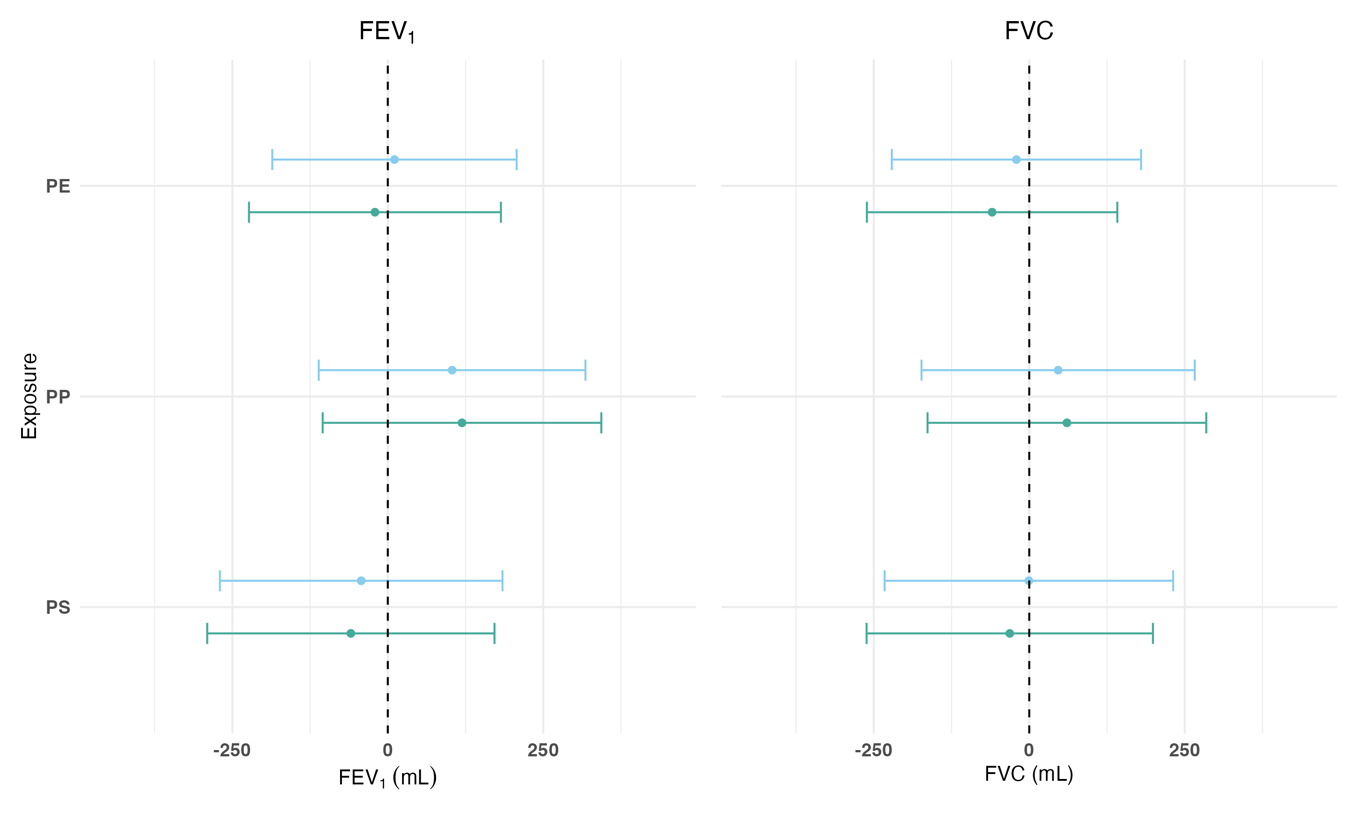


Effect estimates and 95% confidence intervals for binary MNP exposure variables. Minimally adjusted model (blue) adjusted for age, height, weight, and sex. Fully adjusted model (green) adjusted for age, height, weight, sex, recent respiratory infection, smoking, participant education, highest parental education. X axis indicates the regression coefficient and 95% CI for the exposure on FEV_1_ and FVC in mL.

Figure S11 Association between MNPs and Immune Markers presented as mean difference (95% confidence interval) for an IQR increase in MNP exposure, asthmatic and complete case sensitivity analysis

1. Continuous MNP exposures


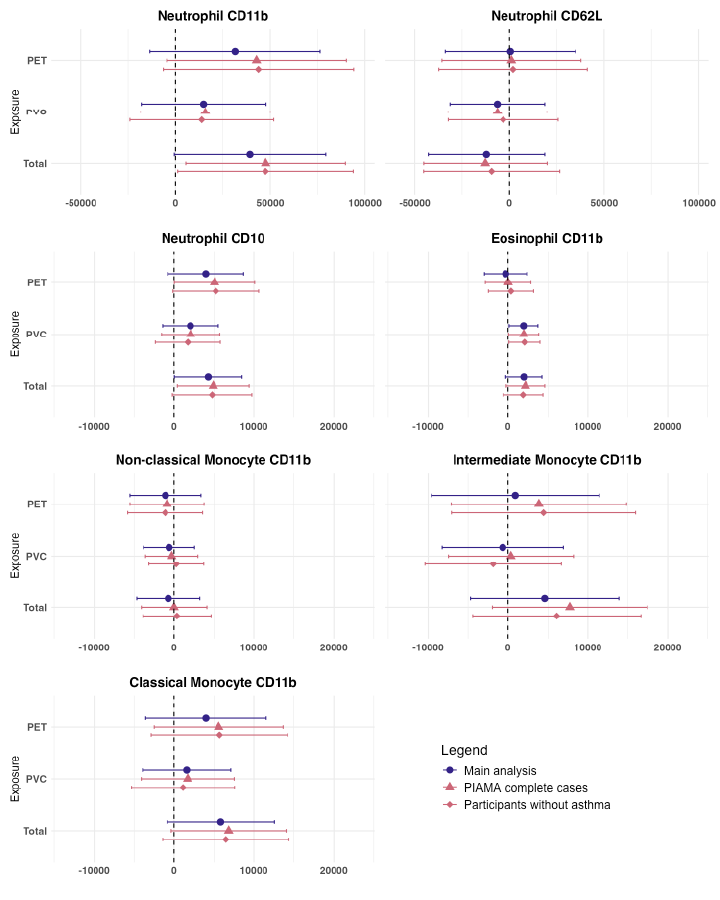


1. Binary MNP exposures


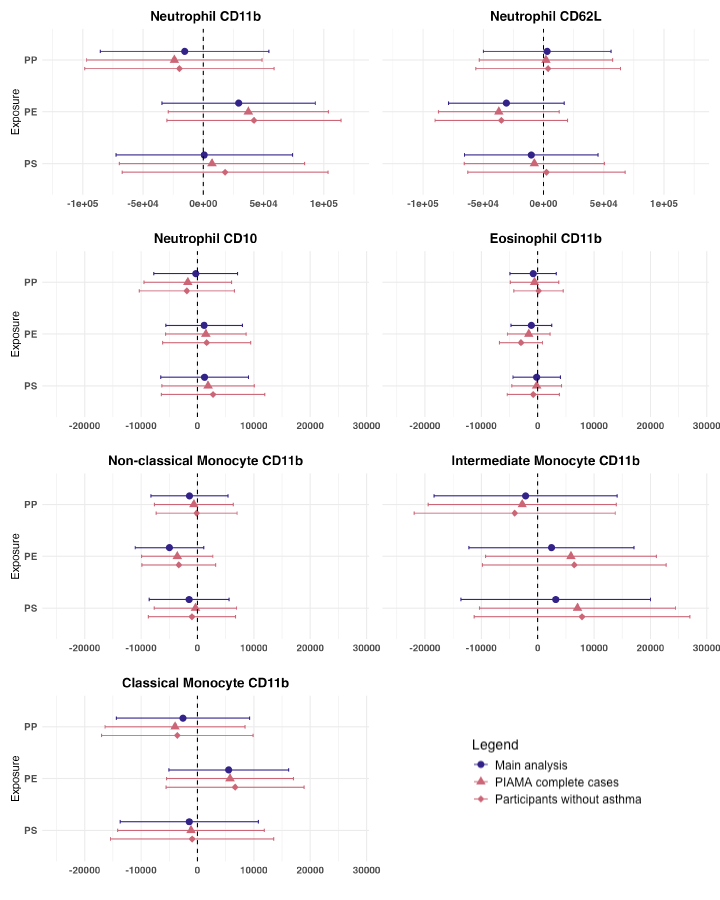


Effect estimates and 95% confidence intervals for MNP exposure variables. Associations with continuous exposures are presented for interquartile range (IQR) increase in exposure. All results presented for fully adjusted models. Main analysis (purple circle), PIAMA complete cases (pink triangle), Participants without asthma (pink diamond). X axis indicates the regression coefficient and 95% CI of the Median Fluorescence Intensity (MFI).

Figure S12. Association between MNPs and Lung Function presented as mean difference (95% confidence interval) for an IQR increase in MNP exposure, asthmatic and complete case sensitivity analysis

1. Continuous MNP exposures


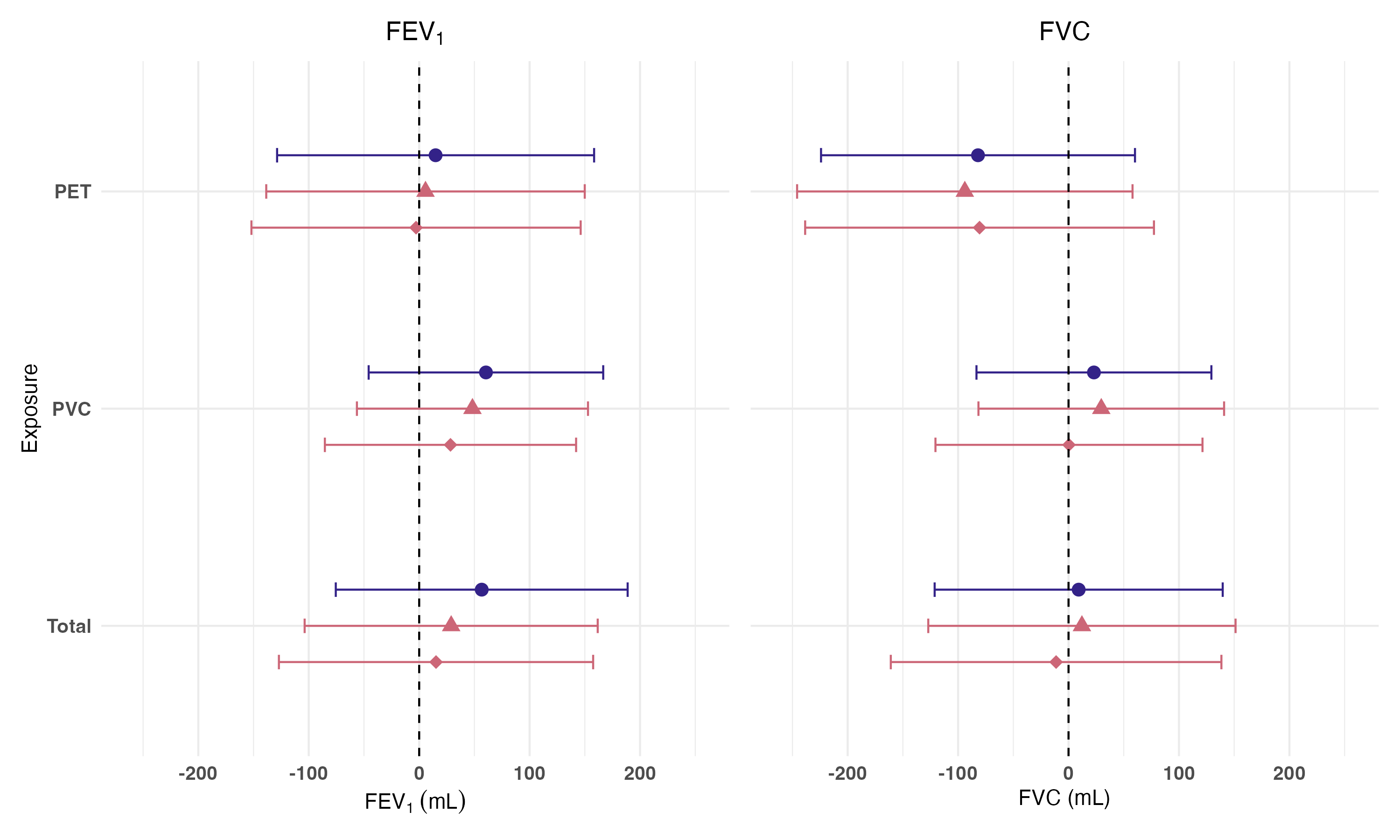


1. Binary MNP exposures


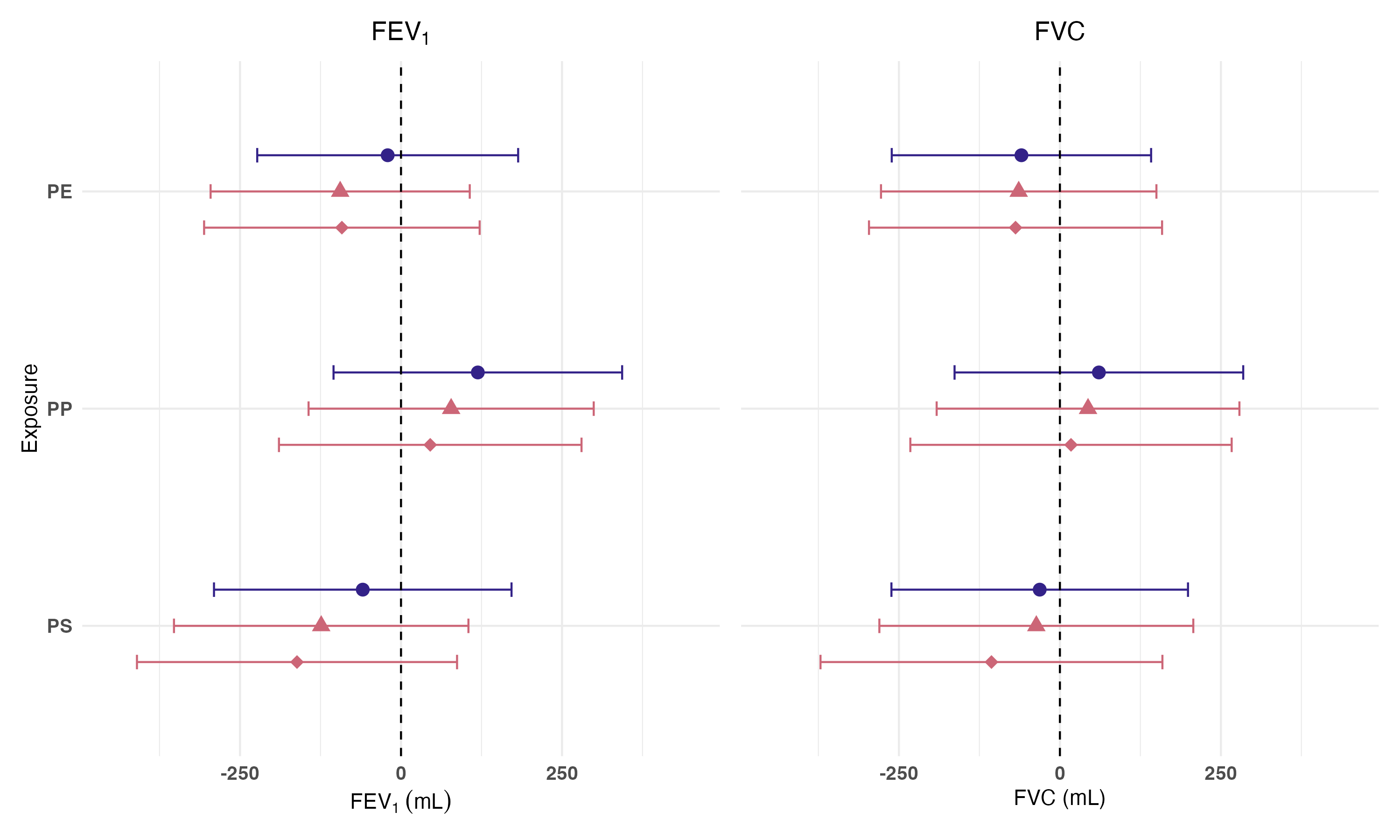


Effect estimates and 95% confidence intervals for MNP exposure variables. Associations with continuous exposures are presented for interquartile range (IQR) increase in exposure. All results presented for fully adjusted models. Main analysis (purple circle), PIAMA complete cases (pink triangle), Participants without asthma (pink diamond). X axis indicates the regression coefficient and 95% CI for the exposure on FEV_1_ and FVC in mL.

Figure S13. Association between MNPs and Immune Markers presented as mean difference (95% confidence interval) for an IQR increase in MNP exposure, exposure assessment sensitivity analysis


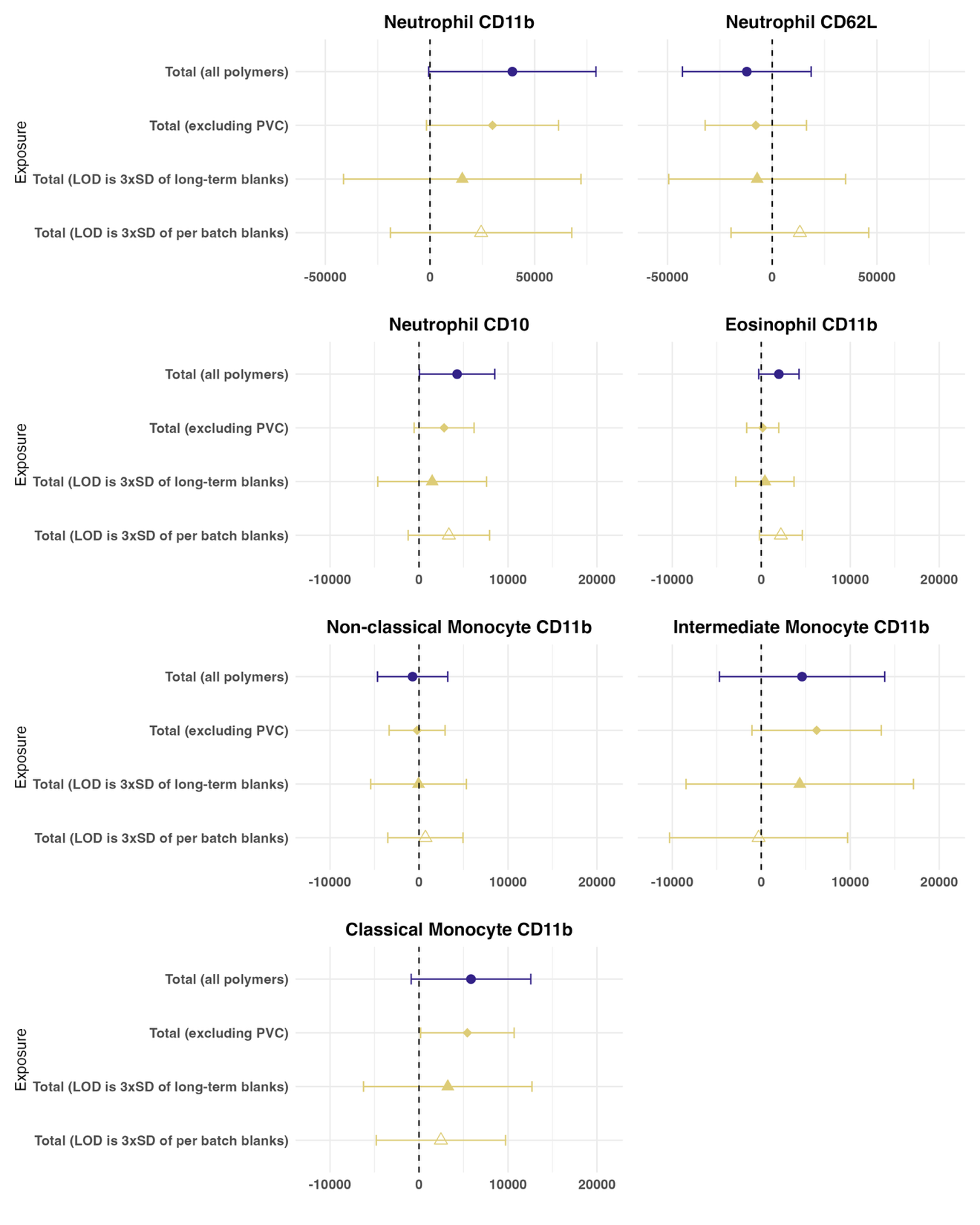


Effect estimates and 95% confidence intervals for MNP exposure variables. Associations with continuous exposures are presented for interquartile range (IQR) increase in exposure. All results presented for fully adjusted models. Main analysis (purple circle), Total MNPs excluding PVC (yellow diamond), Total MNPs when LOD is 3xSD of per batch blanks (solid yellow triangle), Total MNPs when LOD is 3xSD of long-term blanks (open yellow triangle). X axis indicates the regression coefficient and 95% CI of the Median Fluorescence Intensity (MFI).

Figure S14. Association between MNPs and Lung Function presented as mean difference (95% confidence interval) for an IQR increase in MNP exposure, exposure assessment sensitivity analysis


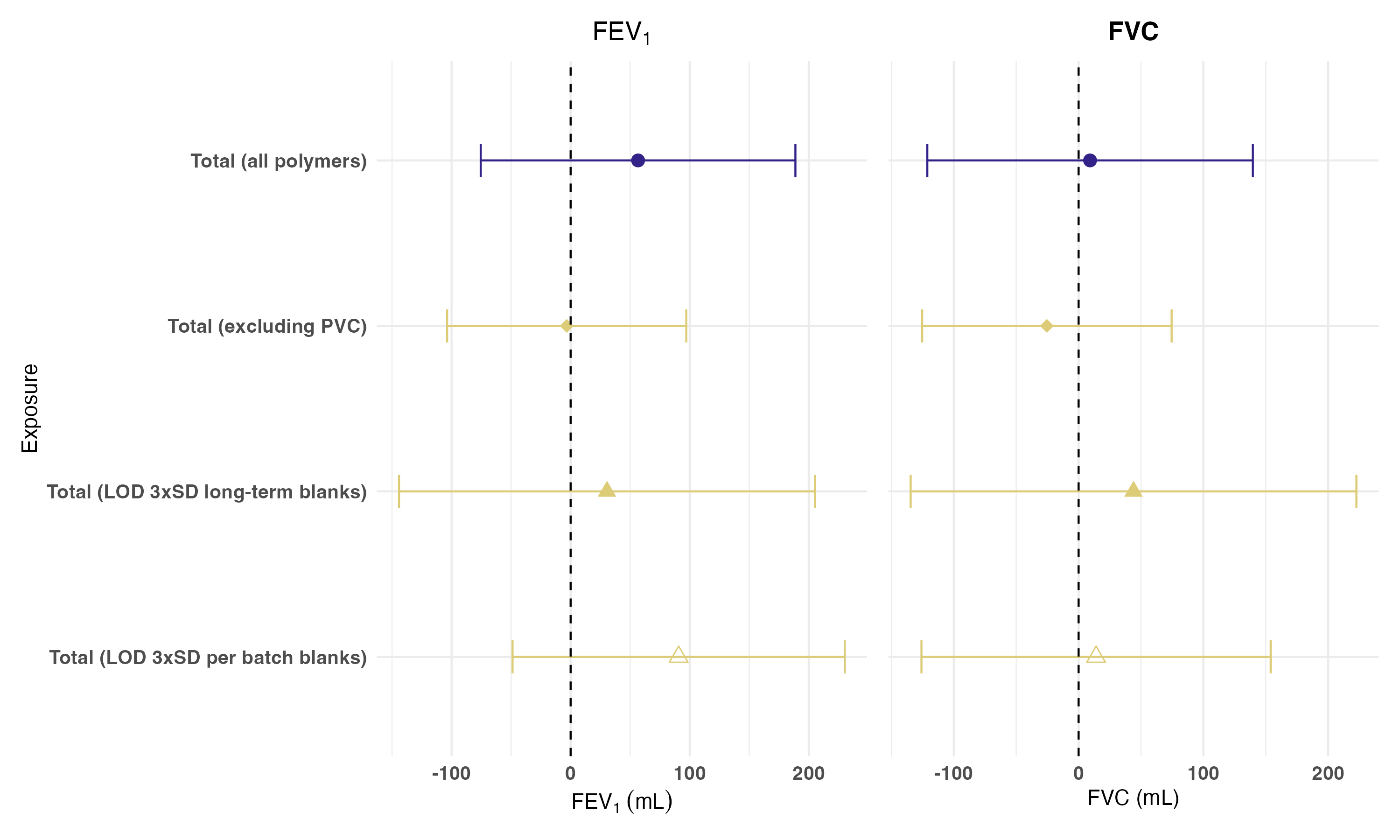


Effect estimates and 95% confidence intervals for continuous MNP exposure variables. Associations are presented for interquartile range (IQR) increase in exposure. All results presented for fully adjusted models. Main analysis (purple circle), Total MNPs excluding PVC (yellow diamond), Total MNPs when LOD is 3xSD of per batch blanks (solid yellow triangle), Total MNPs when LOD is 3xSD of long-term blanks (open yellow triangle). X axis indicates the regression coefficient and 95% CI of the Median Fluorescence Intensity (MFI).

Figure S13. Association between MNPs and Immune Markers presented as mean difference (95% confidence interval) for an IQR increase in MNP exposure, different methods to calculate total MNPs sensitivity analsyis
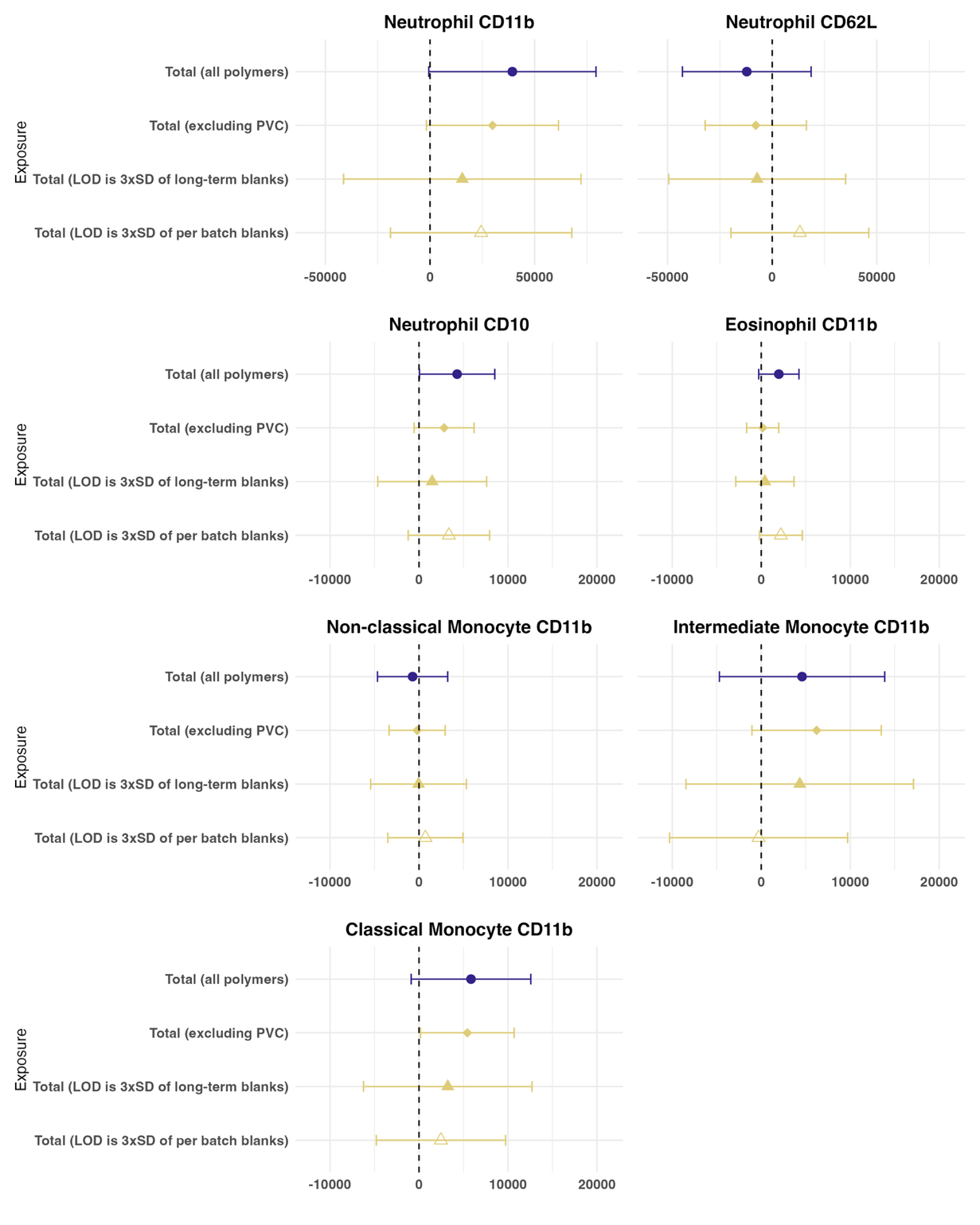


Effect estimates and 95% confidence intervals for MNP exposure variables. Associations with continuous exposures are presented for interquartile range (IQR) increase in exposure. All results presented for fully adjusted models. Main analysis (purple circle), Total MNPs excluding PVC (yellow diamond), Total MNPs when LOD is 3xSD of per batch blanks (solid yellow triangle), Total MNPs when LOD is 3xSD of long-term blanks (open yellow triangle). X axis indicates the regression coefficient and 95% CI of the Median Fluorescence Intensity (MFI).

Figure S14. Association between MNPs and Lung Function presented as mean difference (95% confidence interval) for an IQR increase in MNP exposure, different methods to calculate total MNPs sensitivity analsyis
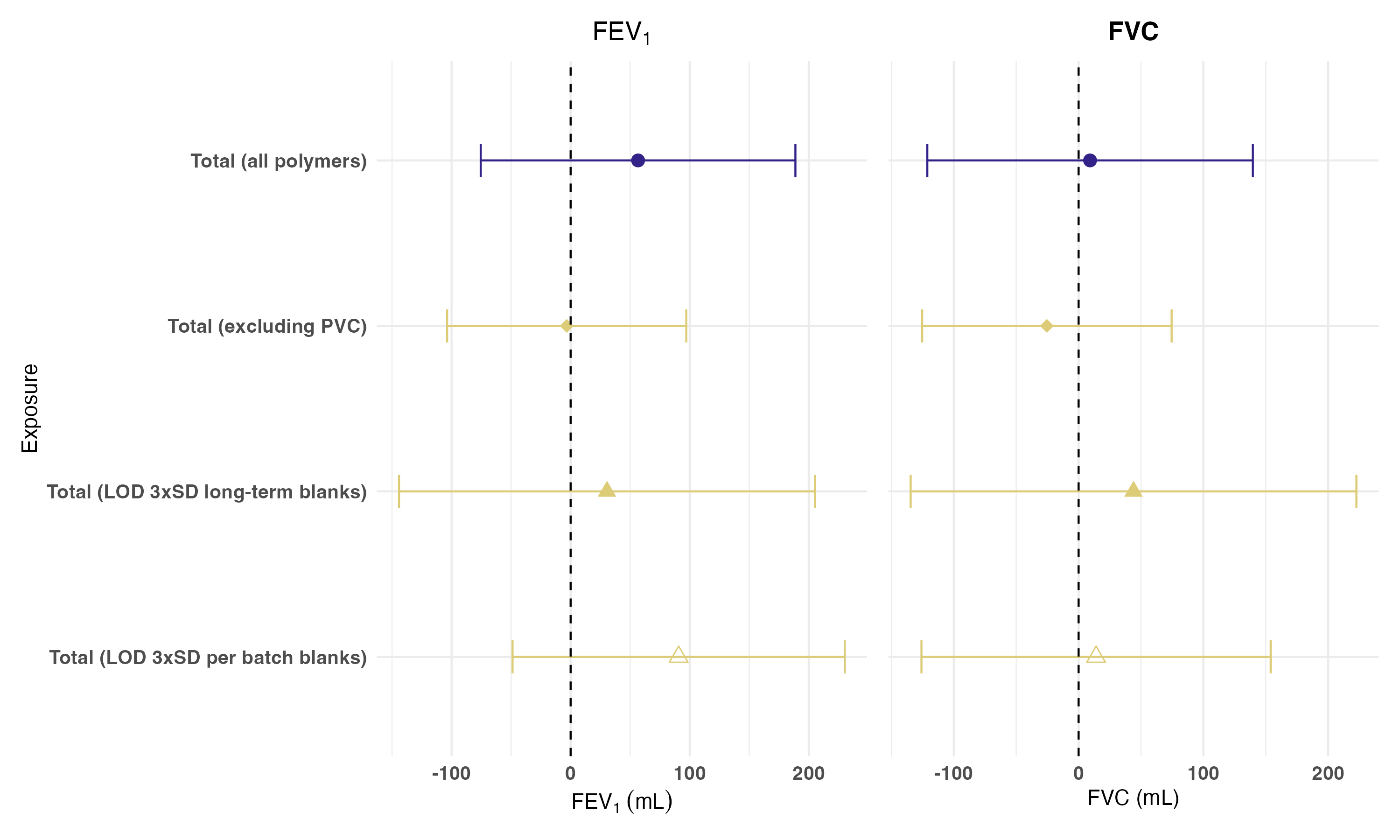


Effect estimates and 95% confidence intervals for continuous MNP exposure variables. Associations are presented for interquartile range (IQR) increase in exposure. All results presented for fully adjusted models. Main analysis (purple circle), Total MNPs excluding PVC (yellow diamond), Total MNPs when LOD is 3xSD of per batch blanks (solid yellow triangle), Total MNPs when LOD is 3xSD of long-term blanks (open yellow triangle). X axis indicates the regression coefficient and 95% CI of the Median Fluorescence Intensity (MFI).

S15. Association between MNPs and Immune Markers presented as mean difference (95% confidence interval) for an IQR increase in MNP exposure when LOD is calculated as three times the standard deviation of the procedural blanks per batch.
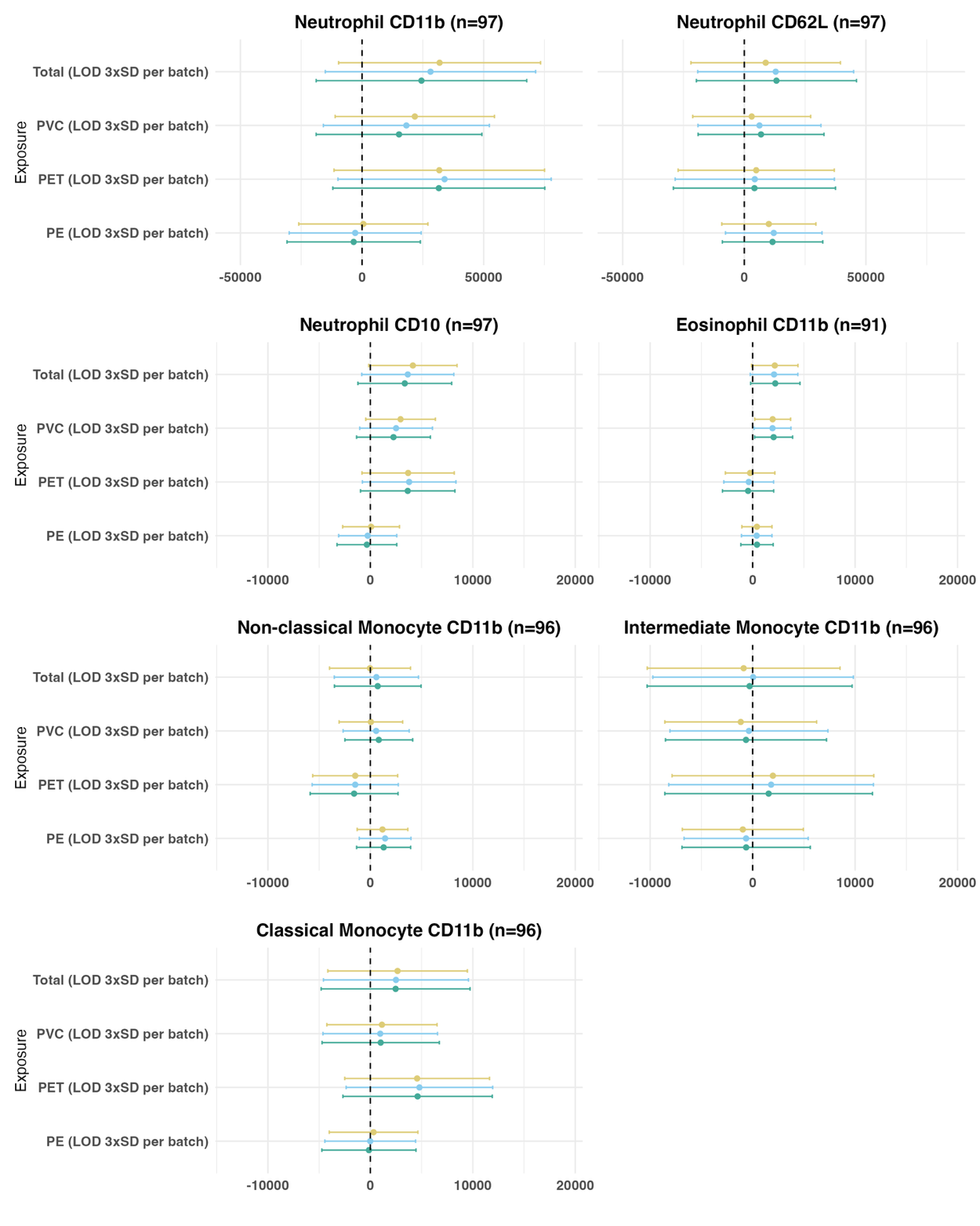


Effect estimates and 95% confidence intervals for continuous MNP exposure variables. Associations are presented for interquartile range (IQR) increase in exposure. Crude model (yellow). Minimally adjusted model (blue) adjusted for age, BMI, and sex. Fully adjusted model (green) adjusted for age, BMI, sex, recent respiratory infection, smoking, participant education, highest parental education. X axis indicates the regression coefficient and 95% CI of the Median Fluorescence Intensity (MFI).

Figure S16. Association between MNPs and Lung Function presented as mean difference (95% confidence interval) for an IQR increase in MNP exposure (n=100) when LOD is calculated as three times the standard deviation of the procedural blanks per batch.


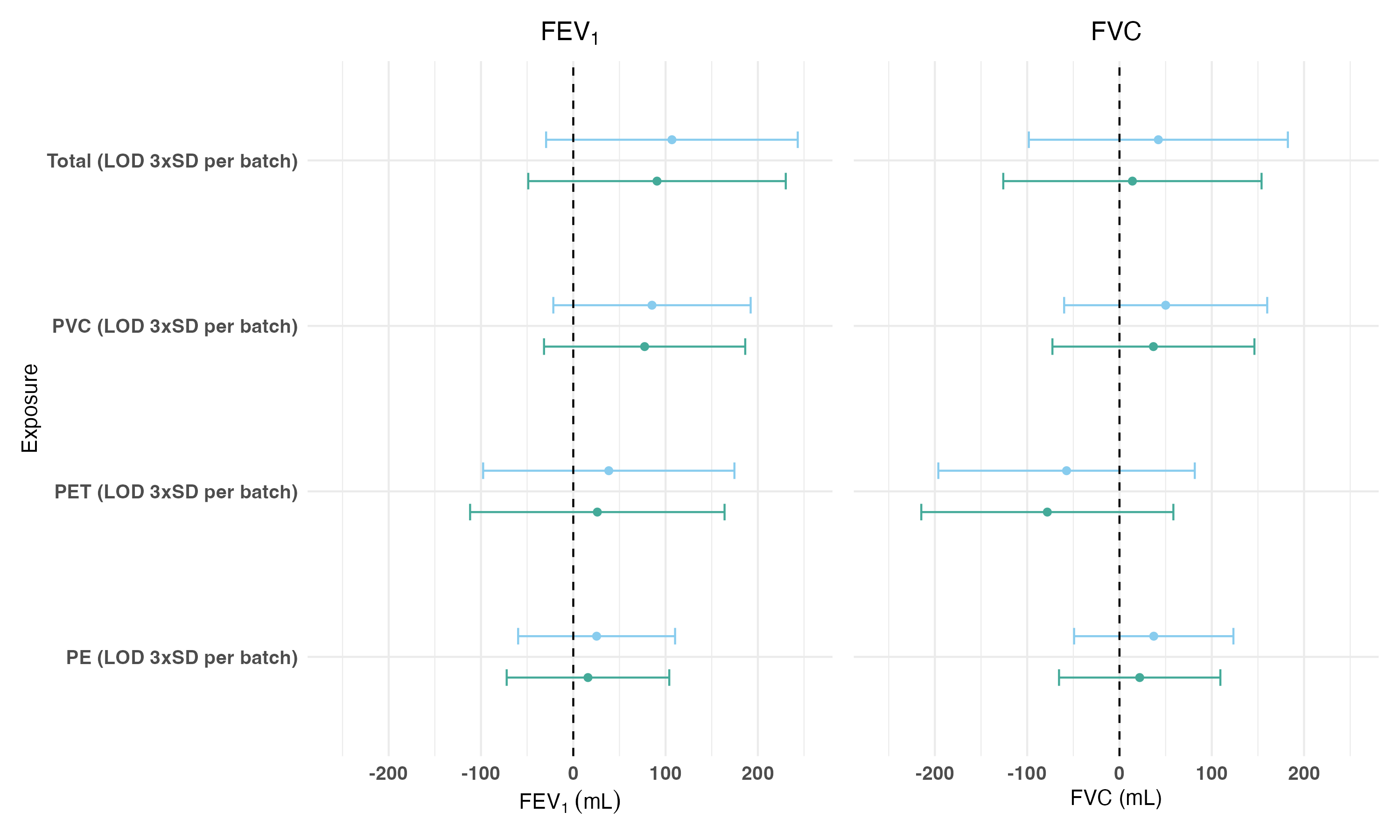
Effect estimates and 95% confidence intervals for continuous MNP exposure variables. Associations are presented for interquartile range (IQR) increase in exposure. Minimally adjusted model (blue) adjusted for age, height, weight, and sex. Fully adjusted model (green) adjusted for age, height, weight, sex, recent respiratory infection, smoking, participant education, highest parental education. X axis indicates the regression coefficient and 95% CI for the exposure on FEV_1_ and FVC in mL.

Figure S17. Association between MNPs and Immune Markers presented as mean difference (95% confidence interval) for binary exposures when LOD is calculated as three times the standard deviation of the procedural blanks per batch.


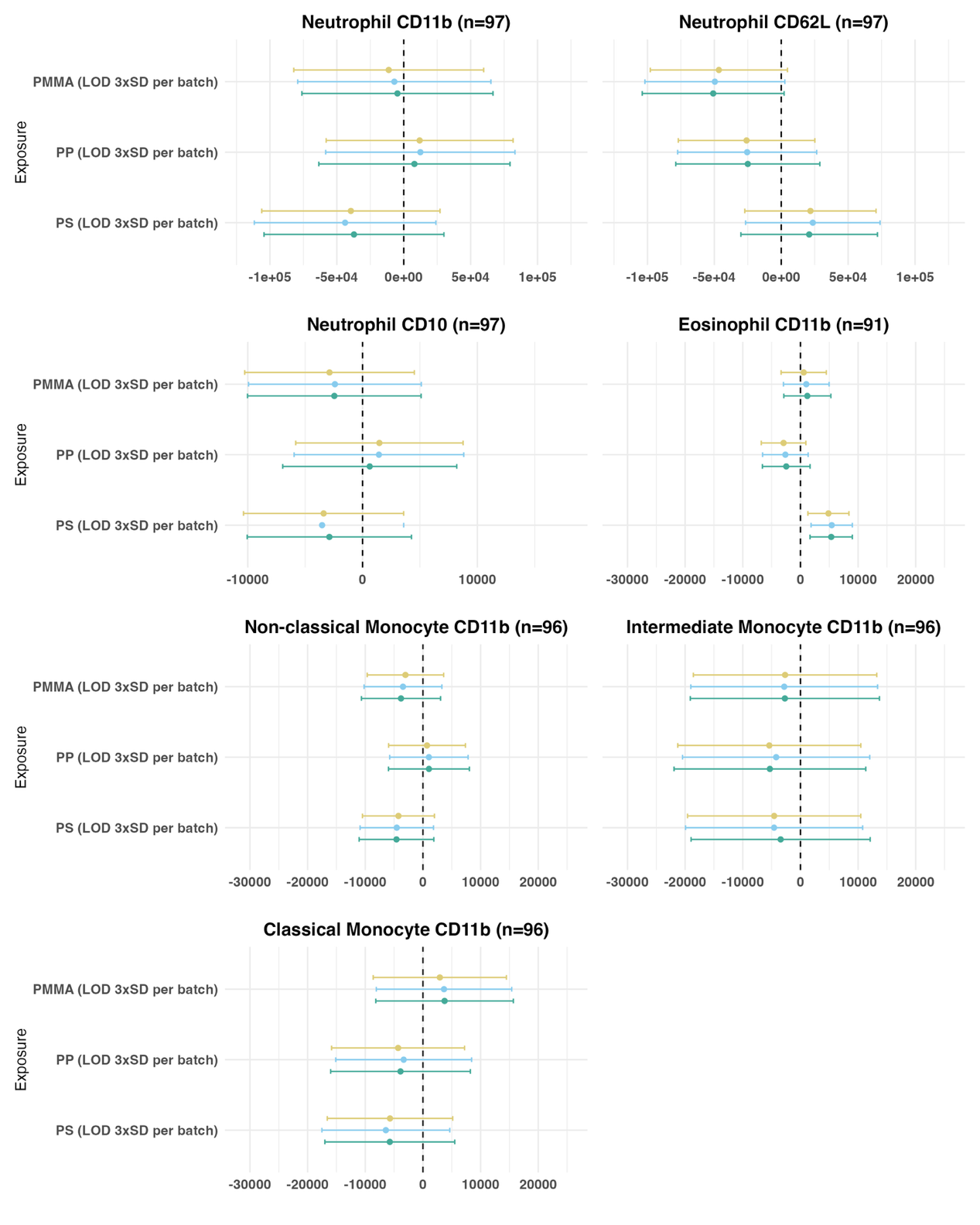


Effect estimates and 95% confidence intervals for binary MNP exposure variables. Crude model (yellow). Minimally adjusted model (blue) adjusted for age, BMI, and sex. Fully adjusted model (green) adjusted for age, BMI, sex, recent respiratory infection, smoking, participant education, highest parental education. X axis indicates the regression coefficient and 95% CI of the Median Fluorescence Intensity (MFI).

Figure S18. Association between MNPs and Lung Function presented as mean difference (95% confidence interval) for an IQR increase in MNP exposure, binary exposures (n=100) when LOD is calculated as three times the standard deviation of the procedural blanks per batch.


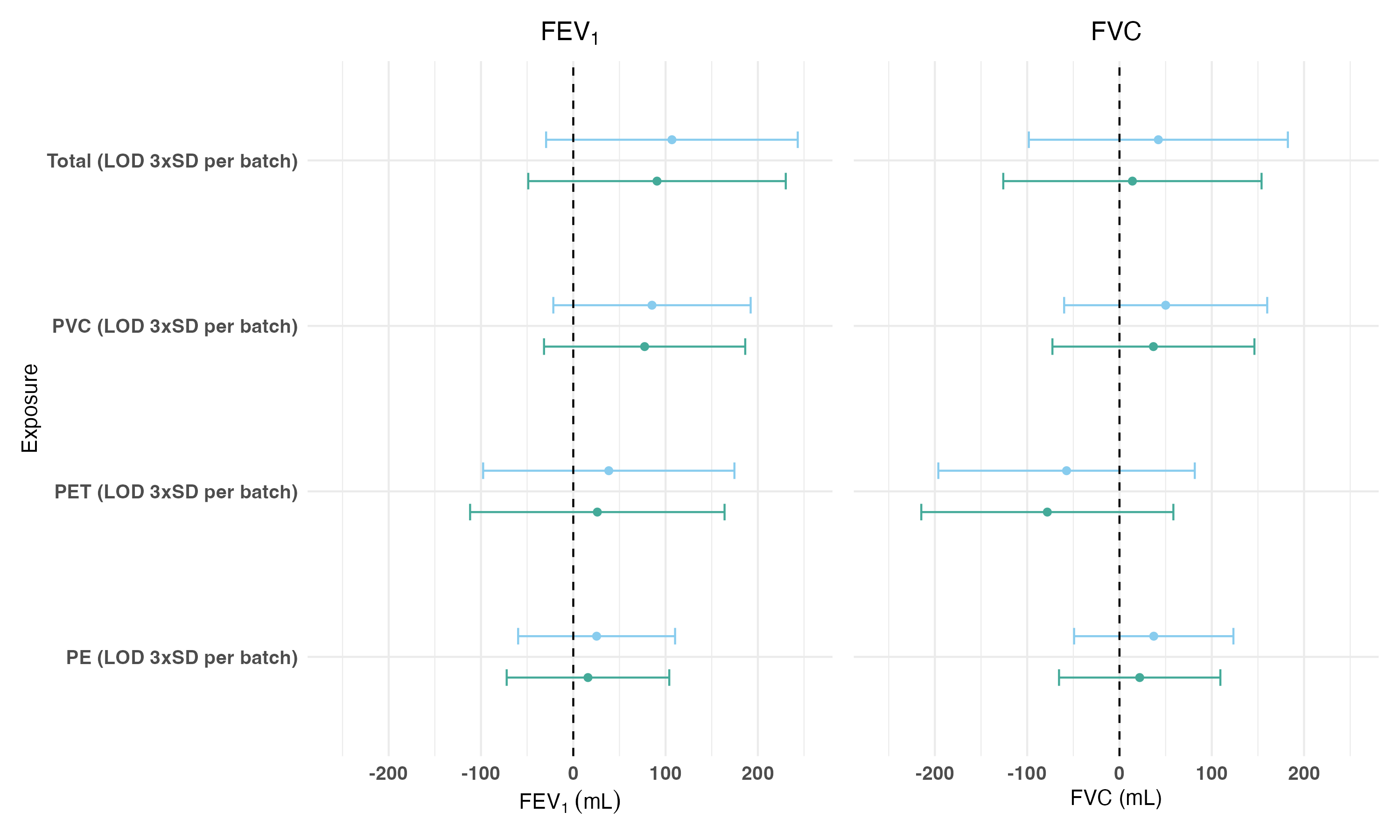


Effect estimates and 95% confidence intervals for binary MNP exposure variables. Minimally adjusted model (blue) adjusted for age, height, weight, and sex. Fully adjusted model (green) adjusted for age, height, weight, sex, recent respiratory infection, smoking, participant education, highest parental education. X axis indicates the regression coefficient and 95% CI for the exposure on FEV_1_ and FVC in mL.

Works Cited

1. de Fraiture EJ, Reniers T, Vreeman NEW, Rettig TCD, van Santvoort HC, Bikker A, et al. Neutrophil phenotypes quantify tissue damage caused by major surgery. Frontiers in Surgery. 2025;Volume 12 - 2025.

2. Durkin A. (2025). Causal relationship between micro- and nanoplastics and lung function. Open Causal. [https://doi.org/10.83031/6lwex6ix](https://eur03.safelinks.protection.outlook.com/?url=https%3A%2F%2Fdoi.org%2F10.83031%2F6lwex6ix&data=05%7C02%7Ca.durkin%40uu.nl%7Cfd95d600c18a4acbd83d08de8431faa8%7Cd72758a0a4464e0fa0aa4bf95a4a10e7%7C0%7C0%7C639093547359130502%7CUnknown%7CTWFpbGZsb3d8eyJFbXB0eU1hcGkiOnRydWUsIlYiOiIwLjAuMDAwMCIsIlAiOiJXaW4zMiIsIkFOIjoiTWFpbCIsIldUIjoyfQ%3D%3D%7C0%7C%7C%7C&sdata=ayKWLFJ69vDmplYcr%2BifFHm7HbayturSNtXEbF9mzhg%3D&reserved=0)
